# Identification of emulsifier potato peptides by bioinformatics: application to omega-3 delivery emulsions and release from potato industry side streams

**DOI:** 10.1038/s41598-019-57229-6

**Published:** 2020-01-20

**Authors:** Pedro J. García-Moreno, Simon Gregersen, Elham R. Nedamani, Tobias H. Olsen, Paolo Marcatili, Michael T. Overgaard, Mogens L. Andersen, Egon B. Hansen, Charlotte Jacobsen

**Affiliations:** 1 National Food Institute, Technical University of Denmark, Copenhagen, Denmark; 2 Department of Chemical Engineering, University of Granada, Granada, Spain; 3 Department of Chemistry and Bioscience, Aalborg University, Copenhagen, Denmark; 4 Department of Bio and Health Informatics, Technical University of Denmark, Copenhagen, Denmark; 5 Department of Food Science, University of Copenhagen, Copenhagen, Denmark

**Keywords:** Biophysical chemistry, Peptides

## Abstract

In this work, we developed a novel approach combining bioinformatics, testing of functionality and bottom-up proteomics to obtain peptide emulsifiers from potato side-streams. This is a significant advancement in the process to obtain emulsifier peptides and it is applicable to any type of protein. Our results indicated that structure at the interface is the major determining factor of the emulsifying activity of peptide emulsifiers. Fish oil-in-water emulsions with high physical stability were stabilized with peptides to be predicted to have facial amphiphilicity: (i) peptides with predominantly α-helix conformation at the interface and having 18–29 amino acids, and (ii) peptides with predominantly β-strand conformation at the interface and having 13–15 amino acids. In addition, high physically stable emulsions were obtained with peptides that were predicted to have axial hydrophobic/hydrophilic regions. Peptides containing the sequence FCLKVGV showed high *in vitro* antioxidant activity and led to emulsions with high oxidative stability. Peptide-level proteomics data and sequence analysis revealed the feasibility to obtain the potent emulsifier peptides found in this study (e.g. γ-1) by trypsin-based hydrolysis of different side streams in the potato industry.

## Introduction

A considerable number of commercial products are oil-in-water emulsions (e.g. food, pharmaceutical, cosmetics)^1^. In addition, aqueous-based food products are enriched with hydrophobic bioactives (i.e., omega-3, vitamins A, D, E, carotenoids, flavonoids or curcumin) by using oil-in-water emulsions as delivery systems^2^. Nevertheless, oil-in-water emulsions are thermodynamically unstable systems. They tend to separate over time into their components (oil and water) due to several physical destabilization mechanisms such as creaming, flocculation, coalescence, and Ostwald ripening^3^. Emulsifiers are the most common stabilizers used in emulsions production since: (i) they facilitate emulsion formation (e.g., by reducing interfacial tension at the oil-water interface), and (ii) they provide physical stability to the emulsion (i.e., by strong steric and/or electrostatic repulsive forces)^4^. Moreover, emulsifiers also have an influence on the chemical stability of emulsions (e.g. oxidative stability) by determining the properties of the oil-water interface (i.e., thickness, porosity, charge, antioxidant activity). Indeed, these interfacial properties play a critical role on the interaction between oil and prooxidants such as radicals, oxygen and trace metals^5^.

Milk proteins such as casein and whey protein are common emulsifiers used for food oil-in-water emulsions due to their excellent functional and antioxidant properties, which lead to physically and oxidatively stable emulsions^6^. Nonetheless, there is an increasing trend to replace animal proteins by plant or microbial proteins in vegetarian or vegan products, as well as to enhance food sustainability^7^. Different approaches have been suggested for the production of sustainable protein-based emulsifiers such as genetic manipulation of microbial systems to obtain designed peptide surfactants. However, costly downstream processing is still a limiting factor for the biological production of peptides^8^. A feasible alternative is to obtain active emulsifier peptides from plant proteins, including side streams from food industry. Interestingly, several plant proteins and especially their hydrolysates (e.g. potato, pulses, algae, soy and RuBisCO proteins) have been reported to show emulsifying properties due to their amphiphilic nature, which permit them to rearrange at the interface and stabilize oil droplets via steric and/or electrostatic repulsions^9–11^.

Particularly attractive are potato proteins, which are recovered from potato juice (a by-product obtained in the extraction of potato starch)^12^. Potato is the world’s fourth most important food crop and it is widely used to extract starch, which makes the potato starch industry an important player in the production of plant-based protein (~240,000 tons/year of potato protein)^13^. Potato proteins are mainly composed of patatins and protease inhibitors^14,15^, both presenting high nutritional and functional properties (e.g. emulsifying)^9,13^. Nevertheless, potato protein is normally extracted by heat/acid precipitation, which results in protein denaturation^16^. Thus, enzymatic hydrolysis is required after extraction to obtain peptides with enhanced solubility and functionality^17^. Interestingly, potato protein hydrolysates have been reported to exhibit not only emulsifying but also antioxidant properties (e.g. due to increased exposure of antioxidant AA side chains)^18^. This is of special interest since potato protein hydrolysates, when used as emulsifiers, could exhibit antioxidant properties at the interface, which is the place where oxidation is initiated in oil-in-water emulsions^19^. In addition, the byproduct from acid precipitation at elevated temperature is protamylasse, which is rich in sugars, organic acids, salts, minerals, free AAs, and, more importantly, peptides and residual proteins (i.e. acid-soluble and heat-stable proteins as well as partially hydrolysed proteins)^20–22^. Although mainly used as fertilizer^23^ and application as growth medium for e.g. cyanophycin production^22^, this self-preserving (due to high dry matter content) side stream may also serve as a potential raw material for isolation of active peptides. More gentle methods for extraction of non-denatured protein from potato fruit juice have been developed^9,24^, although these are generally significantly more expensive and have lower yields than traditional heat/acid precipitation.

To our knowledge, the production of potato protein hydrolysates with functional properties (as well as other protein-based hydrolysates) has only been carried out by a trial-error approach using different proteases to get a wide range of hydrolysates (e.g. with different degree of hydrolysis). Alternatively, in this work, we propose the use of bioinformatics to identify active peptides embedded within the highly abundant proteins present in potato juice. Using bioinformatic-assisted early stage identification of embedded active peptides will make it possible to design a protocol for hydrolysis (e.g. using enzymes with the appropriate specificity) and further purification of the hydrolysate, saving costs and time of extensive screening processes^25^. Therefore, in this work we developed a bottom-up approach where we combined proteomics and bioinformatics to reveal which peptides actually do have functionality, gaining also valuable insight into the underlying molecular properties of peptide emulsifiers. It is worth mentioning that the approach suggested in this work can be applied to any other protein-based raw material, including other types of protein-rich side-streams derived from the food and feed industry.

Therefore, this study aimed at investigating the use of bioinformatics to identify emulsifier peptides within potato proteins. First, based on previous proteomics studies of potatoes (Cv. Kuras)^14,15^, we selected the most abundant proteins present in Kuras in order to create a dataset of interesting protein sequences. Kuras was selected, as it is the predominant cultivar in the northern European potato industry. Secondly, we developed an algorithm in order to predict the emulsifying activity of the peptides depending on their predominant potential conformation at the oil-water interface. Thirdly, we tested the ability of ~40 peptides identified by bioinformatics to have emulsifying activity by evaluating interfacial tension and physical stability of fish oil-in-water emulsions stabilized with these peptides. Peptides with potentially different conformation at oil-water interface (α-helix, β-strand or unordered), as well as different length and charge, were selected and synthesized to gain more insights into the desired properties for emulsifier peptides. Furthermore, for selected potato peptides exhibiting high emulsifying activity, we investigated the ability of these peptides to prevent oxidative deterioration of fish oil-in-water emulsions - a common delivery systems used to fortify food products with long-chain omega-3 fatty acids. Finally, using bottom-up shotgun proteomics, we verified the presence of abundant proteins and predicted peptides in various side streams from industrial potato processing. This data was furthermore used in order to evaluate the feasibility to obtain the selected emulsifier peptides from the side streams by trypsin-based hydrolysis.

## Materials and Methods

### Materials

Synthetic peptides with a purity >70% were purchased from pepMic Co., Ltd (Jiangsu, China). Commercial cod liver oil was kindly provided by Vesteraalens A/S (Sortland, Norway) and stored at −40 °C until use. The fatty acid (%, w/w) content of the fish oil was as follows: C14:0 (0.2%), C16:0 (9.4%), C16:1 n-7 (8.6%), C18:0 (2.0%), C18:1 n-9 (16.2%), C18:1 n-7 (4.6%), C18:2 n-6 (1.8%), C18:3 n-3 (0.1%), C20:1 n-9 (12.6%), C20:5 n-3 (9.1%), C22:1 n-11 (5.9%), and C22:6 n-3 (11.1%). Alpha-, beta-, gamma-, and delta tocopherol contents were 250 ± 2, 0 ± 0, 118 ± 1, 48 ± 1 μg toc/g oil, respectively. The peroxide value (PV) of the fish oil used was 0.12 ± 0.08 meq/kg oil. The characterization of the fish oil used was determined as described elsewhere^26^. Sodium caseinate (MIPRODAN 30) was provided by Arla Foods Ingredients amba (Viby J, Denmark). Protein content of sodium caseinate was reported as 92% (Kjeldal-N × 6.38). Unprocessed potato fruit juice (AKV-PFJ), protamylasse (AKV-K2), and feed-grade protein extract from heat/acid precipitation (AKV-Feed) was supplied by AKV amba (Langholt, Denmark). Protein content was reported as 1.0%, 3.6%, and 76%, respectively (Kjeldal-N × 6.25). Feed-grade protein extract from heat/acid precipitation (KMC-feed) and food-grade protein extract from non-denaturing extraction (KMC-food) were provided by KMC amba (Brande, Denmark). Protein content was reported as 75% and 85%, respectively (Kjeldal-N × 6.25). In a typical season, Kuras constitutes 65% and 45% of the potatoes processed at AKV and KMC, respectively. All other chemicals and solvents used were of analytical grade.

### Bioinformatics

#### Initial potato protein dataset

Based on the studies of Bauw *et al*.^14^ and Jørgensen *et al*.^15^, we selected 9 experimentally determined patatin variants and 25 variants of Kunitz-type protease inhibitors (KTIs) from the Kuras cultivar (Table 1S). We retrieved the AA sequence of each of the proteins from UniProtKB/Swiss-Prot^27^. Then, as only the mature protein is of relevance, the SignalP 4.1 server^28^ was used to predict the presence and location of signal peptides in the proteins, which were subsequently removed. The protein sequences were then cleaved *in silico* into peptides of 7–30 AAs. This decision was due to α-helices having a minimum length of 7 AAs^29^, and large polypeptides (>30 residues) having more complex and unpredictable changes in structure at interfaces than smaller peptides^30^.

#### Identification of emulsifying peptides

The bioinformatics approach used to predict the emulsifying activity of potato derived peptides was based on our previous work^31^, although significant improvements were applied to the algorithm. In principle, the predictions were based on the assumption that a peptide’s emulsifying activity is correlated to the amphiphilic nature of the peptide^32^. Thus, we developed three scores based on peptide amphiphilicity, calculated with the Kyte-Doolittle hydrophobicity scale^33^, and potential different secondary structures of the peptides at the oil/water interface (e.g., α-helix, β-strand and unordered). The algorithm was written in PYTHON 3.6.

For peptides in α-helices, consecutive residues form a 100° angle (5/9 π radians) around the helix central axis. Thus, hydrophobic residues placed alternately three or four residues apart will form a hydrophobic face in the folded peptide. Hydrophilic residues on the opposite face of the helix would result in the helix having a hydrophilic face as well^34^. Therefore, an amphiphilic score for a peptide forming a α-helical structure, was calculated as follows (Eq. 1):

1 $$\alpha =|\mathop{\sum }\limits_{n=1}^{w}K(a{a}_{n})\cdot [\begin{array}{c}\cos (n\cdot \frac{5}{9}\pi )\\ \sin (n\cdot \frac{5}{9}\pi )\end{array}]|$$

where $K(a{a}_{n})$ is the Kyte-Doolittle score of $a{a}_{n}$, $n$ represents the number of the AAs in the peptide sequence and $w$ is the length of the peptide.

Peptides with a β-strand secondary structure have side chains of the AAs pointing alternatively above and below the plane of the β-strand (e.g. every 180° or π radians). This means, that for a β-strand peptide to exhibit emulsifying activity, every second AA should be hydrophobic and hydrophilic, respectively^30^. The amphiphilic score for β-strand forming peptides was calculated as follows (Eq. 2):

2 $$\beta =|\mathop{\sum }\limits_{n=1}^{w}K(a{a}_{n})\cdot [\begin{array}{c}\cos (n\cdot \pi )\\ \sin (n\cdot \pi )\end{array}]|$$

where $K(a{a}_{n})$, $n$ and $w$ represent the same as mentioned for Eq. 1.

Peptides can also display amphiphilic properties by having a hydrophobic and a hydrophilic parts independently of their secondary structure, allowing the peptide to orient itself perpendicularly at the interface^30^. In this study, we have denoted these type of peptides as γ-peptides. The γ amphiphilic score for a peptide with any secondary structure (e.g. α-helix, β-strand or unordered) was calculated as follows (Eq. 3):

3 $$\gamma =|\mathop{\sum }\limits_{n=1}^{k}K(a{a}_{n})-\mathop{\sum }\limits_{m=k+1}^{w}K(a{a}_{m})|$$

where $K(a{a}_{n})$, $n$ and $w$ represent the same as mentioned for Eq. 1. Additionally, $m$ also represents the number of the AA in the peptide sequence and $k$ is the position of the AA which separates the hydrophobic and hydrophilic parts of the peptide.

We introduced two major improvements to the algorithm used in our previous work^31^. First, for the α and β amphiphilic scores, only peptides capable of acquiring the relevant secondary structure (e.g. α-helix or β-strand) were considered. Therefore, the predicted amphiphilic score was set to 0 for α or β peptides with an average probability below 0.3 (thus worse than random) of having α-helix or β-strand conformation, respectively. Secondary structure probabilities of peptides was predicted with NETSURFP-2.0^35^, using the full sequence of the parent protein and then calculating an unbiased average across all AAs within the predicted peptide for acquiring the specified secondary structure. Secondly, as the amphiphilic scores are based on the sum of each AA’s contribution, longer peptides have the potential of achieving higher scores than shorter peptides. In order to adjust for this bias and enable better comparison between peptides with different lengths, a z-score normalization was applied to the raw scores^36^. For each peptide length, we calculated the mean and standard deviation of all scores (α, β and γ) from a set of 40,000 random peptides generated with the web-server RANDSEQ^37^, following the average AA composition computed from the UniProtKB/Swiss-Prot data bank^27^, and with these numbers perform the z-score normalization.

### Functional properties of predicted peptides

#### Emulsifying activity

##### Interfacial tension – pendant drop method

The dynamic interfacial tension of the peptides at the oil-water interface was determined using an automated drop tensiometer OCA20 (DataPhysics Instruments GmbH, Filderstadt, Germany) at 25 °C^38^. Peptide solutions (0.2 wt.%) in 10 mM sodium acetate − 10 mM imidazole buffer (pH 7) were prepared. The peptide solutions were shaken (100 rpm) for 2 h in water bath at 50 °C and overnight at room temperature to allow complete solubilization and rehydration of the peptides (when possible). The peptide solution (water phase) was filled into a syringe with a screwed needle. For each measurement, a small drop of the peptide solution was generated using the automated syringe into a quartz glass cuvette filled with MCT oil (WITARIX MCT 60/40, IOI Oleo GmbH, Hamburg, Germany). The image of the drop was recorded with a camera every 10 s for 30 min. The images were transferred to the drop shape analysis software. The calculation of the interfacial tension was based on the shape analysis of a pendant drop according to the Young-Laplace equation (Eq. 4):

4 $$\Delta {\rm{P}}={\rm{\gamma }}\cdot (\frac{1}{{R}_{1}}+\frac{1}{{R}_{2}})$$

where ΔP (mN/m^2^) is the pressure difference across the interface, γ (mN/m) is the interfacial tension and R_1_ and R_2_ (m) are the principal radii of curvature of the pendant drop. Measurements were carried out in duplicate.

##### Production of emulsions

Emulsions were produced as reported in our previous work^31^. A total amount of 2 g of 5 wt.% fish oil-in-water emulsion stabilized with 0.2 wt.% potato peptides was produced. Peptides (0.2 wt.%) were dissolved in 10 mM sodium acetate – 10 mM imidazole buffer (pH 7). For that, the peptide solutions were shaken (100 rpm) for 2 h in water bath at 50 °C and overnight at room temperature to allow complete solubilization and rehydration of the peptides (when possible). Primary homogenization was done by adding the fish oil to the peptide-buffer solution and mixing at 18,000 rpm for 30 s by using a POLYTRON PT1200E (Kinematic Inc., New York, USA). Secondary homogenization was done using a MICROSON XL2000 sonicator equipped with a P1 probe (Misonix, Inc., New York, USA). Emulsions were homogenized at an amplitude of 75% (maximum amplitude of 180 μm), running 2 passes of 30 s with a break of 1 min between passes. During sonication the emulsions were surrounded by iced water to minimize the increase in temperature. Emulsions stabilized with sodium caseinate (MIPRODAN 30), milk protein exhibiting excellent emulsifying activity, were also produced as control. All emulsions were produced and tested in triplicate.

##### Physical stability of emulsions

###### Droplet size and creaming

The droplet size distribution of the emulsions was measured by laser diffraction in a MASTERSIZER 2000 (Malvern Instruments, Ltd., Worcestershire, UK) at days 0, 1, 3 and 6 during storage in dark at room temperature (~22 °C)^31^. Each emulsion was diluted in recirculating water (3000 rpm), until it reached an obscuration of 12%. The refractive indices of sunflower oil (1.469) and water (1.330) were used as particle and dispersant, respectively. Results are given in volume mean diameter (D_4,3_). Measurements were made in triplicate. Due to the low volume of emulsions produced, creaming was determined qualitatively by observing the appearance of the emulsions. Peptides forming emulsions with small droplet sizes and no creaming were selected for further study.

###### Zeta potential

The zeta potential of the emulsions was measured after one day of storage in a ZETASIZER NANO ZS (Malvern instruments Ltd., Worcestershire, UK) with a DTS1070 cell at 25 °C^31^. Before analysis, the emulsions were diluted (10 μL emulsion in 5 mL buffer). The zeta potential range was set to −100 to +50 mV and the samples were analyzed with 100 runs. Measurements were done in triplicate.

##### Protein modelling and peptide visualization

In order to determine localization of the selected predicted peptides within their mature form protein of origin, we modelled the protein structure of Patatin-B2 (UniProt AC# P15477) for α-10, α-12, γ-1, and γ-36 (QMEAN -0.75); Kunitz-type protease inhibitor, KTI-A (UniProt AC# Q3S488) for β-27 (QMEAN −0.62); KTI-B protein (UniProt AC# Q3S474) for β-22, γ-38, and γ-40 (QMEAN -1.26); and KTI-B protein (UniProt AC# Q3S477) for γ-49 (QMEAN -1.54). Models were built with The SWISS-MODEL WORKSPACE^39^ using the X-ray crystal structure of Patatin-17^40^ (SMTL ID 4pka.1.A), Aspartic Protease Inhibitor 11^41^ (STML ID 5dzu.1.A), and Kunitz-type Proteinase Inhibitor P1H5^42^ (STML ID 3tc2.1.A) as structural templates, respectively, where the latter was the best template and consequently used for all KTI-B-derived peptides. Subsequently, models were visualized in The PyMOL Molecular Graphics System version 1.5.0 (Schrödinger, LLC.). For visualization of individual peptides with visible side chains, residues were colored according to the Swiss-Model hydrophobicity scale (Very hydrophilic residues in blue (R, K, D, E, N, Q, H), partially hydrophilic in purple (P, Y, W, S, T, G), partially hydrophobic in pink (A, M, C, F), and very hydrophobic in red (L, V, I)). Figures containing multiple peptides/proteins visualized with PyMOL were created with INSKAPE version 0.92.3 (Inkscape project).

**Figure 1:**
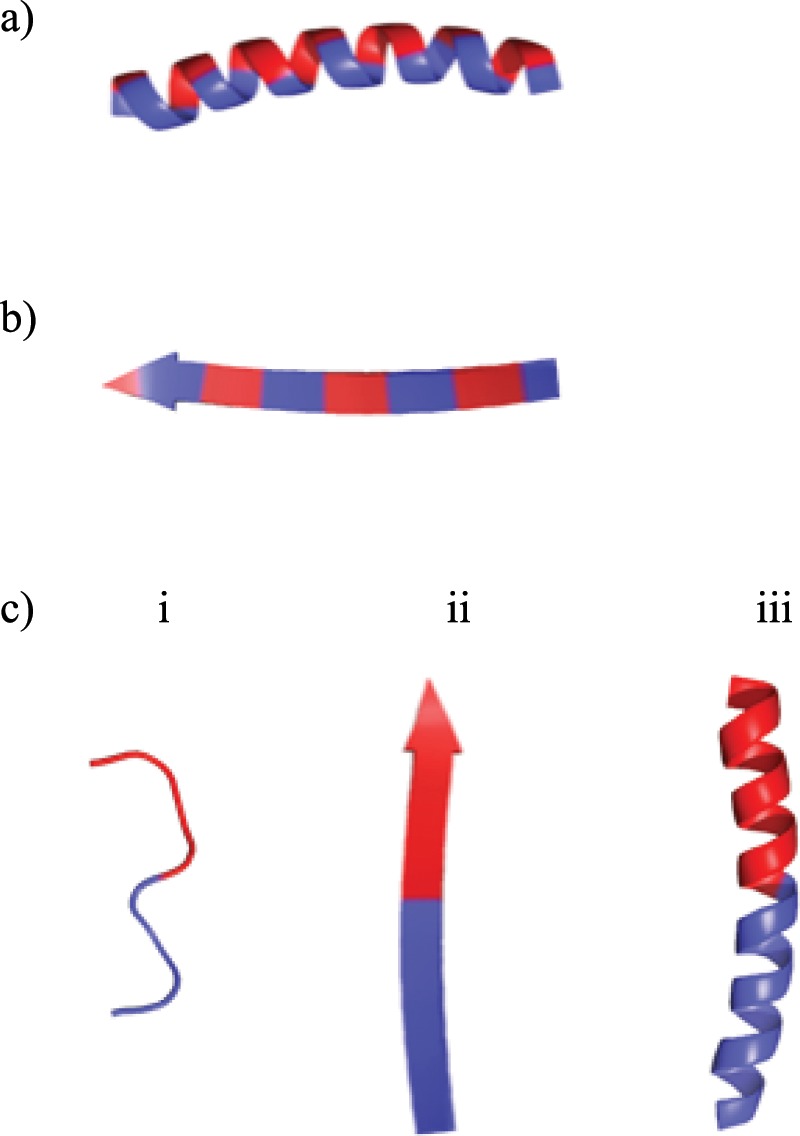
Emulsifiers peptides with facial amphiphilicity: (**a**) α–helix, and (**b**) β–strand; or (**c**) with defined hydrophilic and hydrophobic regions (e.g. a half) in their sequence (γ-peptides: (i) unordered, (ii) β-strand and (iii) α-helix). Figure 1 was created with PyMOL, Version 1.8.4.0. (https://sourceforge.net/projects/pymol/files/pymol/1.8/).

#### Antioxidant activity

##### Oxidative stability of the emulsions

Oxidative stability of emulsions prepared with selected peptides was studied by electron spin resonance (ESR)^31^. ESR analysis was carried out using a benchtop MINISCOPE MS 5000 ESR spectrometer (MAGNETTECH GMBH, Germany). The instrument settings were as follows: magnetic field 320–360 mT, sweep time 60 s, modulation 0.2 mT and frequency 100 kHz. Emulsions were produced as described in section 2.3.1.2 and PBN was added as an ethanol solution (concentration of 50 mg/mL) to have 30 mM of PBN in the oil phase of the emulsion. The oxidative stability of the emulsions was determined during 6 days storage at 20 or 37 °C and samples were measured at days 0, 1 (only for 37 °C), 2, 4 and 6. For each sampling point, emulsion samples (50 μL) were transferred to borosilicate capillary tubes before they were introduced to the cavity of the ESR. Analyses were performed in triplicate at room temperature.

##### *In vitro* antioxidant activity of peptides

Peptides were dissolved in 10 mM sodium acetate - 10 mM imidazole buffer (pH 7) at 0.2 wt.%. 1 1,1-Diphenyl-2-picrylhydrazyl (DPPH) radical scavenging activity, iron (Fe^2+^) chelating activity and ferric (Fe^3+^) reducing power were measured as described elsewhere^26^.

### Proteomics analysis

#### 1D SDS-PAGE analysis and in-gel tryptic digestion

All side stream samples from potato starch processing plants were solubilized with 2% SDS in 200 mM ammonium bicarbonate (pH 9.5) to a final protein concentration (based on provided protein content) of 2 mg/mL. All samples were vortexed for 2 min and sonicated for 30 min. Subsequently, samples were centrifuged at 4,000 g for 15 min to precipitate insoluble proteins and solids. SDS-PAGE of the different potato protein samples was performed on 4–20% gradient gels (GenScript, Piscataway, NJ, USA) under reducing conditions according to manufacturer guidelines by loading 20 µg protein in SDS sample buffer containing dithiothreitol (DTT) to a final concentration of 40 mM (denatured at 95 °C for 5 min). As molecular weight marker, PIERCE Unstained Protein MW Marker P/N 26610 (ThermoFisher Scientific, Rockford, IL, USA) was used. Protein visualization was achieved with Coomassie Brilliant Blue G250 staining using a CHEMDOC MP Imaging System (Bio-Rad, Hercules, CA, USA). Proteins were digested in-gel according to Shevchenko *et al*.^43^ and Fernandez-Patron *et al*.^44^. Briefly, each gel lane was excised and divided into 6 fractions using the MW marker as guide (<14 kDa; 14–25 kDa; 25–45 kDa; 45–66 kDa; 66–116 kDa;>116 kDa). All fractions were cut into 1×1 mm pieces prior to washing, reduction (DTT), alkylation (iodoacetamide), and finally digestion with sequencing grade modified trypsin (Promega, Madison, WI, USA). Digested peptides were extracted, dried down, and resuspended in 0.1% (v/v) trifluoroacetic acid (TFA), 2% acetonitrile (ACN) (v/v). Peptides were desalted using StageTips^45,46^, dried down, and finally resuspended in 0.1% (v/v) TFA, 2% ACN (v/v) for analysis.

#### LC-MS/MS analysis

Tryptic peptides were analyzed by an automated LC–ESI–MS/MS, as previously reported^47^, with modifications to LC system, column supplier, loading flow rate, gradient, isolation window width, survey scan range disabled lock mass, maximum ion injection times, and automated gain control. The system consists of an EASY-nLC system (Thermo Scientific, Bremen, Germany) on-line coupled to a Q Exactive mass spectrometer (Thermo Scientific) via a Nanospray Flex ion source (Thermo Scientific). Tryptic peptides were loaded onto a reverse phase (RP) Acclaim PEPMAP NANOTRAP column (C18, 100 Å, 100 μm. × 2 cm, nanoViper fittings (Thermo Scientific)) in solvent A (0.1% TFA (V/V)) followed by separation on a RP ACCLAIM PEPMAP RSLC analytical column (C18, 100 Å, 75 μm. × 50 cm, nanoViper fittings (Thermo Scientific)). Samples were loaded at a flow rate of 8 μL/min and eluted at a constant flow rate of 300 nL/min during a 120 min ramped gradient, ranging from 5 to 100% of solvent B (0.1% formic acid (FA), 90% (V/V) acetonitrile). Detailed overview of the ramped gradient can be found in Table 2S. The tryptic peptides were directly sprayed through a Distal Coated SilicaTip emitter (FS360–20–10-D-20, 10 µm Tip i.d., PICOTIP (New Objective, Woburn, MA, USA)) into the mass spectrometer. Prior to data acquisition, the mass spectrometer was externally calibrated using PIERCE LTQ VELOS ESI Positive ion calibration solution (Thermo Scientific,), resulting in a sub 5 ppm mass accuracy (R=70,000 at 200 m/z). The mass spectrometer was operated in the positive ion mode, and MS data were acquired using a data-dependent top 12 method, in which the mass spectrometer automatically switched between MS and MS/MS selecting the (up to) 12 most abundant precursors. Only precursors with assigned charge ≥2 were selected for HCD fragmentation using an isolation window of 1.6 Th. Survey scans (400–1200 m/z) were acquired at a resolution of 70,000 at 200 m/z and the HCD spectra were acquired at a resolution of 17,500 at 200 m/z. Lock mass was disabled and the apex trigger window was set at 2–45 s. The maximum ion injection time was set to 100 ms for MS and 75 ms for MS/MS scans. The automatic gain control for MS and MS/MS was 1e6 and 1e5, respectively. The minimum percentage of the target value at maximum injection time, the underfill ratio, was set to 3.5%. A dynamic exclusion of 30 s was used for minimizing repetitive selection of the same ions. Normalized collision energy was set to 30 eV. For reassigning the monoisotopic mass based on the precursor isotope pattern, the features “peptide match” and “exclude isotopes” were enabled.

#### Analysis of proteomics data

Protein identification and quantification was performed using MAXQUANT 1.6.0.16^48,49^. The sequence database for potatoes (*Solanum tuberosum*) were retrieved from UniProt^27^ (tax: 4113). Standard setting were employed including a false discovery rate (FDR) of 1% on both peptide and protein level. Reversed sequences were used as decoys to control FDR and common contaminants were included in the searches. Protein quantification was done using both unique and razor peptides. General sample statistics can be found in Table 3S. Each sample was analyzed as 6 fractions with matching between runs and dependent peptides enabled to boost identification rates. For in-sample relative protein quantification, the iBAQ algorithm was used. iBAQ quantifies proteins by summing all MS1 peptide intensities for a given protein (group) and dividing it by the number of theoretical tryptic peptides (between 6 and 30 AAs). iBAQ intensities were normalized to the sum of all iBAQ intensities after removal of reverse hits and contaminants, thereby providing the relative iBAQ (riBAQ) which can be regarded as an estimate of the relative molar distribution of proteins (groups).

Following initial analysis, three fragment (length <75% of related full length mature proteins) database entries (Q9S8K2 (2.3 kDa KTI fragment), P24744 (2.9 kDa KTI fragment), and Q2MYE7 (14.9 kDa patatin fragment)) were identified with high riBAQ thereby providing an unwanted bias into the protein distribution. The database was subsequently manually curated and the fragments removed, as all identified fragment-related peptides were also covered by full length protein entries in the database. Data was reprocessed with the curated database and no fragment entries were identified with high riBAQ (>0.5% riBAQ).

#### Abundance of proteins and selected peptides in potato processing side streams

Using proteomics data from all samples, a list of abundant identified proteins (>1% riBAQ in any sample) was created as a supplement to the list of initially selected proteins. All identified proteins with riBAQ> 1% were processed in the prediction algorithm.

Peptide-level abundance was estimated using protein level abundance. All identified proteins containing the exact sequence of a predicted peptide (based on multiple sequence alignment (MSA) using CLC Sequence Viewer 8.0 (Qiagen Bioinformatics) were grouped and the sum of riBAQs was used as a measure to estimate maximum relative molar recovery of the peptide in each side stream.

### Statistical analysis

STATGRAPHICS 18 (Statistical Graphics Corp., Rockville, MD, USA) was used for data analysis as previously described^31^. Data were expressed as mean ± standard deviation. One-way ANOVA was carried out. Firstly, multiple sample comparison analysis was performed to identify significant differences between samples. Secondly, mean values were compared by using the Tukey’s test. Differences between means were considered significant at p < 0.05.

## Results and Discussion

### Prediction of emulsifying potato peptides by bioinformatics

Peptides can exhibit emulsifying properties through different structural conformations (Fig. 1). For instance, the facial amphiphilicity of α-helix or β-strand structures (Fig. 1a,b) implies that the peptides can anchor at the interface with its hydrophobic face projected into the non-polar oil-phase and the hydrophilic face projected into the polar aqueous-phase^32,50^. On the other hand, γ-peptides exhibiting perpendicular amphiphilicity (e.g. unordered, α-helix or β-strand) (Fig. 1c) could adsorb perpendicularly at the interface projecting its hydrophobic and hydrophilic regions to the oil and aqueous phases, respectively^30^.

**Figure 2:**
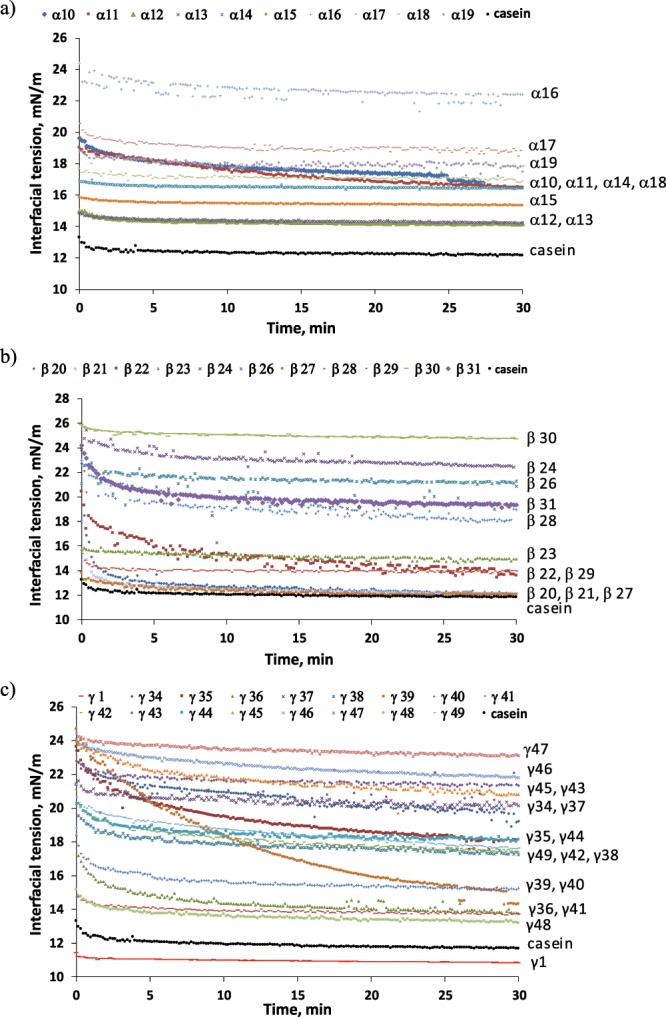
Interfacial tension at the MCT oil-aqueous phase interface, with the aqueous phase containing 0.2 wt.% peptides in 10 mM sodium acetate - 10 mM imidazole buffer (pH 7): (**a**) α-peptides, **(b**) β-peptides, and (**c**) γ-peptides. The bare MCT oil-water interfacial tension was 26 mN/m.

Using the previously experimentally identified abundant potato proteins of Bauw *et al*. (2006) and Jørgensen *et al*. (2006), 9 patatin and 25 KTI proteins from *Solanum tuberosum* (see Table 1S in Supplementary Material) were used in this study to identify emulsifying peptides using bioinformatics. Both patatins^51^ and KTIs^52^ are highly structured proteins, and hence, there is a potential of finding emulsifying peptides with different conformations at the oil/water interface, embedded within patatin and KTI sequences.

Based on the proposed bioinformatics approach, 38 peptides were predicted to exhibit emulsifying properties (Table 1). The group consists of 10 α-peptides, 11 β-peptides and 17 γ-peptides with an amphiphilic score higher than 2 (Table 1). The nomenclature of the peptides refers to the equation used to predict their score (e.g., the α equation was used to predict the emulsifying power of peptide α-10). It is worth mentioning that the 38 selected peptides present different characteristics (e.g. length and pI) (Table 1). This will increase the options to up-grade different proteins found in potato juice, as well as to gain more insights on how secondary structure, length and charge of the peptides influence physical and oxidative stability of the emulsions they produce. Peptides with>70% similarity were clustered and resultantly, some of the peptides studied originate from the same cluster. These peptides have a high identity core with varying N and/or C terminal extension. This was the case for: (i) α-peptides, α-10, α-11 and α-12; α-13 and α-14; α-15 and α-16; α-17, α-18 and α-19; (ii) β-peptides, β-20 and β-21; β-23, β-24 and β-27; (iii) γ-peptides, γ-34 and γ-35; γ-36 and γ-37; γ-38, γ-39 and γ-49; γ-42 and γ-43; γ-44 and γ-45 (Table 1). This indicates that there are specific regions within both patatins and KTIs (across different protein isoforms) that have a specifically high potential for emulsifying activity.

**Table 1:** Properties of potato peptides predicted by bioinformatics to have emulsifying activity.

Peptide	Sequence	Score*	AA Separation**	Accession Number(s)	Number of residues	α-helical content, %***	β-strand content, %***	Purity****, %	Mw, g/mol	pI*****	Net charge***** (pH 7)
α-10	KKPVSKDSPETYEEALKRFAKLLSDRKKL	2.176	—	P15477 P11768 Q42502 Q3YJT3	29	26	7	92.7	3405.9	10.38	4
α-11	EALKRFAKLLSD	3.522	—	P15478 P15477 Q3YJT5 P11768 Q2MY60 Q2MY43 Q42502 Q3YJT3	12	—	—	97.0	1390.6	9.92	1
α-12	DSPETYEEALKRFAKLLSD	3.037	—	P15477 P11768 Q42502 Q3YJT3	19	74	5	94.9	2212.4	4.16	−2
α-13	NRPFAAAKDIVPFYFEHGPHIFN	2.155	—	P15477 P11768 Q42502 Q3YJT3	23	—	—	95.1	2688.0	7.53	0.2
α-14	AKDIVPFYFEHGPHIFN	2.255	—	P15477 P11768 Q42502 Q3YJT3	17	—	—	89.5	2031.3	6.05	-0.8
α-15	IPATILEFLEGQLQEVDNN	2.000	—	P15477 P11768 Q42502 Q3YJT3	19	—	—	86.3	2143.4	0.61	−4
α-16	ILEFLEGQLQEVDN	2.978	—	P15478 P15477 P11768 Q42502 Q3YJS9 Q3YJT3	14	—	—	83.7	1646.8	0.61	−4
α-17	KYDGKYLMQVLQE	2.029	—	P15478 Q3YJT5 Q2MY60 Q2MY43 Q3YJS9	13	—	—	89.8	1614.9	6.67	0
α-18	KYLMQVLQEKLGE	2.022	—	P15478 Q3YJT5 Q2MY60 Q3YJS9	13	—	—	96.8	1578.9	6.77	0
α-19	KYLMQVLQEKL	2.058	—	P15478 Q3YJT5 Q2MY60 Q3YJS9	11	—	—	74.5	1392.7	9.49	1
β-20	ELDSRLSYRIISTFWGALGGDVYLGKSPN	2.630	—	Q3S474 A0A097H149 Q66LL8 Q66LL2	29	—	—	75.0	3215.6	6.87	0
β-21	ELDSRLSYRIISTFWGALGGDVYL	3.131	—	Q3S474 A0A097H149 Q66LL8 Q66LL2	24	—	—	70.0	2732.0	4.17	−1
β-22	CPFSSDDQFCLKVGV	2.501	—	Q3S474 A0A097H149 L0AQL9 Q2XPX8 Q2XPY0	15	13	33	78.9	1644.9	3.71	−1.1
β-23	FIPLSTNIFEDQLLNIQFNIPT	2.391	—	Q3S488 Q3S482 Q3S479 H9B8I8	22	—	—	89.9	2577.9	0.69	−2
β-24	LNIQFNI	4.066	—	Q8RXA3 Q3S489 Q3S488 Q3S481 Q38M77 Q3S482 Q3S479 H9B8J1 H9B8I9 H9B8I8 H9B8J0	7	—	—	95.3	861	3.66	0
β-26	GKELDPRLSYRI	2.218	—	Q3S477 Q2XPX8 Q2XPY0 Q8LJQ0	12	—	—	86.9	1446.7	9.73	1
β-27	LNIQFNIPTPKLC	2.213	—	Q3S488 Q8RXA3 Q3S482 Q3S479 H9B8J1	13	23	31	70.6	1500.8	8.84	0.9
β-28	VHQNGKRRLALVKDNPLDVSFK	2.056	—	Q3S474 A0A097H149 Q2XPX8 Q2XPY0	22	—	—	94.2	2534.9	10.92	3.1
β-29	IGSSSHFGPHIFEGELLNIQFDIS	2.027	—	Q3S477 Q2XPX8 Q2XPY0 Q8LJQ0	24	—	—	84.7	2644.9	4.3	−2.8
β-30	DDNFCAKVGVVIQ	2.016	—	Q3S489 Q3S488 Q3S481 Q38M77 Q3S479 H9B8I9	13	—	—	80.3	1407.6	3.71	−1.1
β-31	LGGDVYLGKSPNSDAPCP	2.011	—	Q8RXA3 Q3S488 Q3S481 Q38M77 Q3S482 Q3S479 H9B8I9 H9B8I8 H9B8J0	18	—	—	91.8	1789.9	3.71	−1.1
γ-1	GIKGIIPAIILEFLEGQLQEVDNNKDAR	4.146	14	P15477 P11768	28	75	0	99.4	3094.5	4.2	−2
γ-34	CRDDNFCAKVGVVI	3.387	5	Q3S488 Q3S481 Q38M77 Q3S489 Q3S479 H9B8I9	14	—	—	89.0	1538.8	5.9	−0.1
γ-35	RDDNFCAKVGVVI	3.933	4	Q3S488 Q3S481 Q38M77 Q3S489 Q3S479 H9B8I9	13	—	—	87.8	1435.7	6.05	−0.1
γ-36	FDVIGGTSTGGLLTAMITTPNENNRP	3.190	17	P15477 Q3YJT5 P11768 Q2MY60 Q42502 Q3YJT3	26	31	23	78.6	2676.9	3.93	−1
γ-37	LLTAMITTPNENNRP	4.037	6	P15477 Q3YJT5 P11768 Q2MY60 Q42502 Q3YJS9 Q3YJT3	15	—	—	77.6	1684.9	6.86	0
γ−38	FCLKVGVVHQNGKRRLALVKDNP	3.168	8	Q3S474 Q2XPX8 A0A097H149 Q2XPY0	23	0	65	89.7	2592.1	10.91	4
γ-39	HQNGKRRLALV	4.790	7	Q3S474 Q3S477 Q2XPX8 A0A097H149 Q2XPY0 Q66LL8 Q66LL2 Q8LJQ0	11	—	—	98.3	1291.5	12.13	3.1
γ-40	SSDDQFCLKVGVV	3.137	5	Q3S474 Q2XPX8 A0A097H149 Q2XPY0 L0AQL9	13	0	46	80.6	1396.6	3.71	−1.1
γ-41	KDNPETYEEALKRFAKLLS	3.066	13	P15478 Q3YJT5 Q2MY60 Q2MY43	19	—	—	70.5	2252.5	6.88	0
γ-42	DTNGKELNPNSSYRIISIGRGALGGDVYL	3.055	14	Q3S481 Q38M77 Q3S489 H9B8I8 H9B8J0	29	—	—	79.0	3080.4	6.89	0
γ-43	NPNSSYRIISI	3.401	7	Q3S481 Q38M77 Q3S489 H9B8I8 H9B8J0	11	—	—	98.6	1263.4	9.41	1
γ-44	DNFCAKVGVVIQNGKRR	3.044	11	Q3S488 Q3S481 Q3S489 Q3S479 H9B8I9	17	—	—	72.3	1904.2	10.68	2.9
γ-45	VGVVIQNGKRR	4.779	5	Q8RXA3 Q3S488 Q3S481 Q3S489 Q3S482 Q3S479 H9B8J1 H9B8I9 H9B8J0	11	—	—	94.6	1225.5	12.13	3
γ-46	FAKLLSDRKKLRANK	3.043	5	P15478 P15477 P11768 Q2MY60 Q2MY43 Q42502 Q3YJS9 Q3YJT3	15	—	—	95.2	1788.2	11.57	5
γ-47	TPNENNRPFAAAKDIV	3.024	8	P15477 Q3YJT5 P11768 Q42502 Q3YJT3	16	—	—	97.3	1756.9	6.62	0
γ-48	GIIPATILEFLEGQLQEVDNN	3.003	11	P15478 Q42502 Q3YJS9 Q3YJT3	21	—	—	92.2	2313.6	0.61	−4
γ-49	FCLKVGVIHQNGKRRLALVK	2.478	8	Q3S477 Q8LJQ0	20	0	75	99.0	2279.8	11.49	5

^*^Predicted amphiphilic score.

**AA used by the algorithm to define the divide between the hydrophobic and hydrophilic part of the peptide.

***Fraction of residues located in a given secondary structure conformation (within the native protein) as implied by homology modelling. This was carried out only for the 9 selected peptides.

****Purity of the synthetic peptides used as received from pepMic Co., Ltd (Jiangsu, China).

*****pI and net charge were calculated by using peptide property calculator from INNOVAGEN (Innovagen AB, Lund, Sweden).

### Functional properties of peptides: emulsifying and antioxidant

Emulsifier peptides must comply with the following requirements: (i) rapid adsorption to the surface of the newly formed oil droplets during homogenization, (ii) anchoring and possibly conformation change at the oil/water interface causing a reduction of interfacial tension, and (iii) formation of an adequate interfacial layer which prevents droplet aggregation (e.g. by coalescence)^53^.

#### Emulsifying activity

##### Interfacial tension and physical stability of emulsions

The Gibbs free energy (ΔG) required to increase the contact area (ΔA) between oil and water is proportional to the interfacial tension at the oil-water interface (γ) (ΔG = γ·ΔA) (McClements, 2004). Emulsifiers adsorb at the oil-water interface shielding the oil molecules from the water molecules, which reduces interfacial tension. Therefore, it is interesting to investigate the ability of peptides to reduce interfacial tension since it determines the possible increase in contact area (e.g. oil droplets break up) for a constant value of energy provided during homogenization^54^. Once adsorbed to the surface of the newly created oil droplets, emulsifier peptides need to form an interfacial film that provides steric hindrance and/or electrostatic repulsive forces, which prevent droplets aggregation and thus physical destabilization of the emulsion^1^. Hence, peptides reducing interfacial tension at the oil-water interface do not always show high emulsifying activity (e.g. if they do not properly rearrange its structure at the interface leading to a continuous and/or thick interfacial layer; and/or if they do not provide electrostatic repulsion between droplets)^32^. Therefore, the emulsifying activity of the identified potato peptides by bioinformatics was further investigated by determining the physical stability of 5 wt.% fish oil-in-water emulsions stabilized with 0.2 wt.% of peptides at pH 7.

Figure 2 shows the evolution of the interfacial tension at the MCT oil-peptides solution interface. Table 2 shows information on the solubility of the peptides employed as well as pH, droplet size, and physical stability during storage of the emulsions stabilized with the predicted peptides. Among the 38 peptides tested, 14 peptides were not totally soluble resulting in cloudy aqueous phases (e.g. no transparent solutions were obtained and white peptides particles could be observed). The obtained emulsions had a pH ranging from 5.6 to 7. Differences in pH are due to the acidic impurities present in the synthetic peptides used, and to the insufficient capacity of the buffer employed to keep pH constant at 7. Nonetheless, sodium acetate-imidazole buffer was preferred to phosphate buffer in order to avoid interferences of phosphate buffer in oxidative stability studies as consequence of its chelating activity.

**Figure 3:**
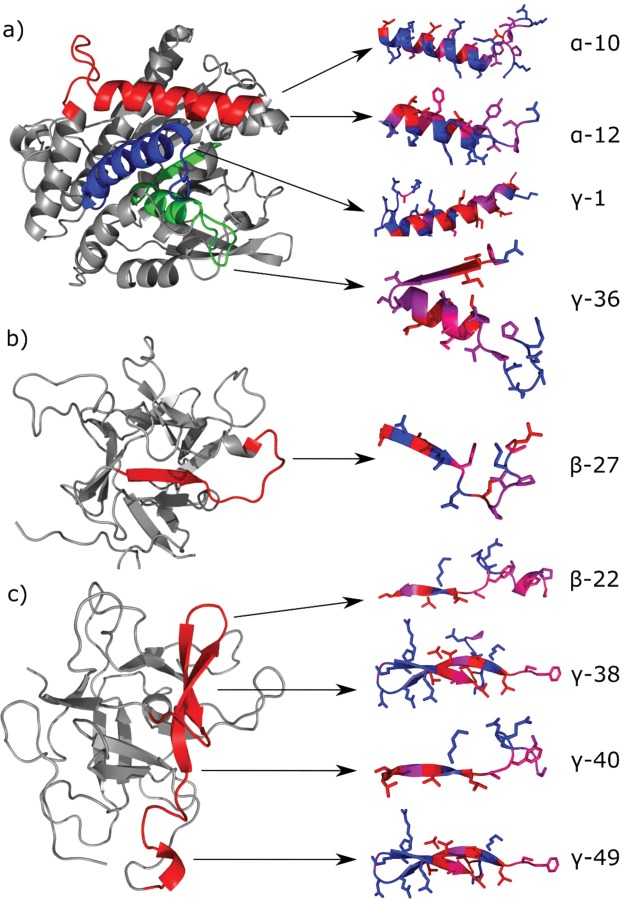
Left: Localization of predicted emulsifier peptides based on template homology modelling using SWISS-MODEL Workspace^39^ (https://swissmodel.expasy.org/) and visualized in PyMOL version 1.5.0 (https://pymol.org/2/). Models are made using the protein of origin for the predicted peptides (Table 1). (**a**) Patatin-B2 (UniProt AC# P15477) was modelled using the X-ray structure of Patatin-17 (SMTL ID 4pka.1.A) as template, showing localization of the overlapping α-10 and α-12 (red), γ-1 (blue), and γ-36 (green). (**b**) KTI-A (UniProt AC# Q3S488) was modelled using the X-ray structure of Aspartic Protease inhibitor 11 (SMTL ID 5dzu.1.A) as template, showing localization of β-27 (red). (**c**) KTI-B (UniProt AC# Q3S474) was modelled using the X-ray structure of Kunitz-type protease inhibitor P1H5 (SMTL ID 3tc2.1.A) as template, showing localization of the overlapping β-22, γ-38, and γ-40 (red). The model for γ-49 (UniProt AC# Q3S477) using P1H5 (SMTL ID 3tc2.1.A) is superimposable with the model for the remaining KTI-B-derived peptides (i.e. γ-49 has the same structure) and is shown on the same model for simplicity. Right: Secondary structure of predicted peptides (as found within the models for their proteins of origin) with visible side chains and residue coloring according to the Swiss-Model hydrophobicity color scale (most hydrophobic residues in red and most hydrophilic residues in blue). Individually visualized proteins/peptides were assembled into the final figure using INKSCAPE version 0.92.3 (https://inkscape.org/).

**Table 2:** Values of pH, droplet size, zeta potential and observations on creaming of the emulsions.

Emulsion	Solubility of peptides*	pH	D_4,3_ (μm)**				Zeta potential (mV)	Observations***		
			Day 0	Day 1	Day 3	Day 6		Day 0	Day 1	Day 6
α-10	Soluble	6.3	1.9 ± 1.3^a^	1.5 ± 0.6^a^	4.3 ± 0.6^a^	5.5 ± 0.4^b,*^	28.0 ± 2.1^e^	1	1	1
α-11	Soluble	6.5	40.4 ± 10.6^c^	60.9 ± 8.2^b^	—	—	−27.6 ± 4.6^c^	4	4	4
α-12	Soluble	6.3	0.3 ± 0.02^a^	2.3 ± 0.8^a^	2.7 ± 1.0^a^	3.0 ± 0.5^a,b,*^	−54.8 ± 3.5^b^	1	1	1
α-13	Soluble	6.5	94.9 ± 6.1^d^	111.6 ± 33.1^c^	—	—	−26.3 ± 0.7^c,d^	4	4	4
α-14	Soluble	6.1	16.3 ± 1.2^b^	11.8 ± 1.9^a^	11.8 ± 0.1^b^	12.8 ± 3.3^c,ns^	−15.5 ± 3.8^d^	2	2	3
α-15	Soluble	5.6	0.3 ± 0.07^a^	0.3 ± 0.06^a^	0.3 ± 0.07^a^	0.3 ± 0.07^a,ns^	−69.1 ± 3.9^a^	1	3	3
α-16	Soluble	5.7	1.0 ± 0.9^a^	0.3 ± 0.09^a^	0.4 ± 0.0^a^	0.4 ± 0.0^a,ns^	−67.0 ± 3.5^a^	3	3	3
α-17	Cloudy	6.5	9.3 ± 3.0^a,b^	17.4 ± 3.7^a^	34.6 ± 0.7^d^	24.2 ± 0.1^e,*^	−31.2 ± 2.7^c^	2	2	2
α-18	Cloudy	6.1	8.9 ± 0.3^a,b^	12.8 ± 1.7^a^	15.8 ± 1.8^b^	26.0 ± 2.9^e,*^	−46.8 ± 6.9^b^	2	2	2
α-19	Soluble	6.7	21.3 ± 5.5^b^	31.9 ± 4.7^a,b^	22.4 ± 4.0^c^	18.2 ± 1.6^d,ns^	−24.0 ± 4.0^c,d^	1	2	2
β-20	Cloudy	6.2	14.0 ± 1.5^a,b^	14.8 ± 1.2^a,b^	26.9 ± 4.1^b^	37.6 ± 1.7^c,*^	−30.5 ± 4.2^c,d^	2	2	2
β-21	Cloudy	6.9	24.1 ± 9.7^b^	28.1 ± 4.6^a,b,c^	38.6 ± 2.8^c,d^	42.8 ± 2.1^d,*^	−37.6 ± 3.1^c^	2	2	2
β-22	Soluble	6.1	6.0 ± 0.6^a^	6.1 ± 0.5^a,b^	6.1 ± 0.9^a^	8.5 ± 0.1^a,b,*^	−70.9 ± 3.0^a^	1	1	1
β-23	Cloudy	6.6	16.9 ± 1.7^a,b^	26.5 ± 6.4^a,b,c^	29.9 ± 6.2^b,c^	33.6 ± 0.2^c,*^	−54.0 ± ± 0.9^b^	1	1	1
β-24	Cloudy	6.4	40.6 ± 1.4^c^	41.9 ± 11.6^c^	—	—	−36.2 ± 13.3^c^	4	4	4
β-26	Soluble	6.3	50.1 ± 14.7^c^	98.0 ± 26.5^d^	—	—	−19.2 ± 4.9^d^	4	4	4
β-27	Cloudy	6.3	2.6 ± 0.9^a^	2.4 ± 0.1^a^	4.3 ± 0.5^a^	5.4 ± 1.1^a,*^	15.8 ± 2.9^e^	1	1	1
β-28	Soluble	6.1	6.9 ± 2.3^a^	31.1 ± 0.06^b,c^	40.3 ± 4.8^d^	—	−29.9 ± 2.9^c,d^	2	4	4
β-29	Cloudy	6.3	12.0 ± 0.2^a,b^	15.0 ± 0.9^a,b^	12.9 ± 0.4^a^	10.6 ± 0.9^b,ns^	−28.9 ± 1.1^c,d^	2	2	2
β-30	Cloudy	6.0	10.5 ± 0.1^a,b^	12.4 ± 2.1^a,b^	10.5 ± 0.3^a^	10.7 ± 0.1^b,ns^	−21.8 ± 2.1^d^	2	2	2
β-31	Soluble	6.1	2.9 ± 0.6^a^	6.1 ± 0.6^a,b^	8.7 ± 0.6^a^	12.4 ± 3.5^b,*^	−21.8 ± 0.9^d^	3	3	3
γ-1	Soluble	6.2	0.6 ± 0.2^a^	0.7 ± 0.2^a^	0.6 ± 0.2^a^	0.6 ± 0.2^a,ns^	−61.3 ± 3.1^b^	1	1	1
γ-34	Cloudy	6.0	16.6 ± 1.3^a^	18.4 ± 0.7^d^	20.7 ± 0.7^d^	21.1 ± 0.9^e,*^	−20.2 ± 7.5^e^	2	2	2
γ-35	Cloudy	6.0	24.0 ± 0.0^a,b^	25.6 ± 1.4^e^	34.2 ± 1.0^d^	31.7 ± 1.4^f,*^	−24.5 ± 3.4^d,e^	2	2	2
γ-36	Soluble	6.4	0.8 ± 0.0^a^	3.8 ± 0.8^b^	3.3 ± 0.1^a,b^	2.5 ± 0.2^a,b,*^	−54.5 ± 1.7^b^	1	1	1
γ-37	Soluble	6.1	0.6 ± 0.0^a^	0.5 ± 0.0^a^	0.8 ± 0.5^a^	0.7 ± 0.4^a^	−39.2 ± 1.7^c^	3	3	3
γ-38	Soluble	6.1	5.7 ± 0.1^a^	4.1 ± 0.9^b^	5.7 ± 1.7^b^	8.8 ± 0.1^c,d^	34.6 ± 3.2^g^	1	1	1
γ-39	Soluble	6.8	54.9 ± 0.0^b,c^	—	—	—	−21.5 ± 1.4^e^	4	4	4
γ-40	Cloudy	6.8	1.0 ± 0.1^a^	1.0 ± 0.1^a^	0.9 ± 0.1^a^	0.9 ± 0.1^a,ns^	−55.6 ± 2.2^b^	1	1	1
γ-41	Soluble	6.5	69.8 ± 17.1^c^	—	—	—	−29.4 ± 7.2^c,d,e^	4	4	4
γ-42	Cloudy	6.9	1.3 ± 0.3^a^	3.5 ± 1.8^b^	2.1 ± 0.1^a,b^	7.2 ± 1.5^c,*^	−35.4 ± 1.7^c,d^	1	2	2
γ-43	Soluble	7.0	60.3 ± 41.2^c^	—	—	—	−24.9 ± 5.4d^d,e^	4	4	4
γ-44	Cloudy	7.0	2.4 ± 0.2^a^	6.8 ± 0.7^c^	10.4 ± 4.3^c^	11.0 ± 0.0^d,*^	30.0 ± 1.05^f,g^	1	1	1
γ-45	Soluble	6.8	82.5 ± 10.5^c^	—	—	—	−22.7 ± 2.3^e^	4	4	4
γ-46	Soluble	6.4	87.5 ± 4.5^c^	—	—	—	−20.4 ± 4.4^e^	4	4	4
γ-47	Soluble	6.1	123.7 ± 8.8^d^	—	—	—	−21.5 ± 4.3^e^	4	4	4
γ-48	Soluble	5.9	0.4 ± 0.0^a^	0.7 ± 0.4^a^	2.4 ± 1.4^a,b^	2.4 ± 1.7^a,b,ns^	−78.2 ± 2.9^a^	1	3	3
γ-49	Soluble	6.7	1.7 ± 0.3^a^	0.8 ± 0.0^a^	1.3 ± 0.2^a,b^	4.5 ± 0.3^b,*^	18.9 ± 3.8^f^	1	1	2
Casein	Soluble	6.9	1.1 ± 0.2	1.1 ± 0.2	0.9 ± 0.2	1.0 ± 0.2	−41.5 ± 1.1	1	1	1

^*^Solubility of peptides in 10 mM sodium acetate - 10 mM imidazole buffer (pH 7). Soluble: totally soluble; cloudy: no totally soluble.

**No values (—) were obtained for emulsions with phase separation.

***The number codes stand for: 1: no creaming; 2: creaming; 3: oil layer above the emulsion; 4: phase separation.

For each type of peptides, different letters in the same column indicate significant differences between samples (p<0.05). ‘ns’ indicates not significant differences (p>0.05), whereas ‘*’ indicates significant differences (p<0.05) in D_4,3_ between days 1 and 6 for the same emulsion.

###### α-peptides

In the case of α-peptides, the highest reduction in interfacial tension (i.e. compared to the interfacial tension for the bare MCT oil-water interface, 26 mN/m) was found for predicted emulsifying peptides having above 18 AAs (e.g. α-12, α-13, α-15 and α-10) (Fig. 2a). It correlated well with the physical stability of the these emulsions since α-10, α-12 and α-15, as well as α-16 led to emulsions with the lowest droplet size at day 0 and during storage (Table 2). However, emulsions stabilized with α-15 or α-16 showed an oil layer after one day of storage or after immediately production, respectively (Table 2). This denotes the inability of α-15 and α-16, with α-16 embedded in α-15, to emulsify the total amount of oil used, and thus the superior emulsifying activity of α-10 and α-12. The low emulsifying activity showed by α-16 correlated well with the poor ability of short α-peptides (e.g. <14 AAs, such as α-16, α-17 and α-19) to reduce interfacial tension (Fig. 2a), which may be associated with an insufficient length that decreased amphiphilic α-helicity, and then anchoring of the peptide at the interface. Nevertheless, the same explanation is not valid for α-15, which considerably reduced interfacial tension. Moreover, α-15 had a considerable negative charge (−4 at pH 7), which resulted in a highly negative zeta potential (−69.1 ± 3.9 mV) favoring electrostatic repulsions between droplets, but also potential repulsion between peptides molecules that may result in a low interfacial coverage. In addition, α-15 had the same length as α-12 (19 AAs), which may be sufficient to adopt a potential amphiphilic α-helix structure at the oil/water interface, although this remains to be investigated. On the contrary, the poor emulsifying activity observed for α-13, which exhibited high reduction in interfacial tension (Fig. 2a), cannot be attributed to length (23 AAs) but possibly because of insufficient net charge (0.2 at pH 7) (Table 1). This correlates with the low net zeta potential value obtained for the emulsion stabilized with α-13 (<30 mV) (Table 2), which may could not provide sufficient electrostatic repulsion to avoid coalescence of droplets right after formation (as shown by the high D_4,3_ value at day 0, Table 2). In the same line, Dexter^55^ concluded that a compromise between zeta potential and interfacial binding affinity (both controlled, among other properties, by peptide charge) is desired for optimum emulsifier peptides.

Interestingly, α-11, with the highest amphiphilic score (3.522), and length of 12 AAs, did not decrease interfacial tension to a higher extent when compared to a longer peptide, which shared the same core peptide but had considerably lower score (e.g. α-10 with 29 AAs and score of 2.176) (Fig. 2a). Moreover, α-11 showed a low emulsifying activity, leading to phase separation after emulsion production (Table 2). These results denote that although α-11 had highly hydrophobic and hydrophilic faces and a short length which could favor its diffusion to the interface, there are other main aspects involve in the reduction of interfacial tension by peptides. The structural rearrangements that the peptide is able to undergo at the interface in order to adsorb, e.g. projecting its hydrophobic and hydrophilic regions to the oil and water phases, are of special importance.

Indeed, our results indicated that α-10 and α-12 with positive and negative zeta potential, respectively; and different length (29 and 19 AAs) showed high reduction in interfacial tension (Fig. 2a) and the highest emulsifying activity with D_4,3_<6 μm after six days storage (Table 2). With α-12 being totally embedded in α-10, both peptides have the potential to adopt amphiphilic α-helix structure (see next section), which may facilitate peptide anchoring at the interface and peptide film layer formation, providing physical stability to the emulsion. Altogether, α-helical peptides above a certain length (>18 AAs) appear to have higher potential to adopt the predicted amphiphilic α-helix conformation at the interface when compared to shorter peptides (<14 AAs). Likewise, Enser *et al*.^50^ reported the superior emulsifying power of peptides above 11–15 AAs (e.g. at least three turns of α-helix), which had a more pronounced amphiphilic α-helix conformation in the aqueous phase, and thus potentially also at the interface. Additionally, results reported by Saito *et al*.^32^ indicated higher surface pressure (i.e. higher reduction in interfacial tension when compared to the bare oil-water interface) for a peptide with amphiphilic α-helix conformation when compared to a peptide with a non-amphiphilic α-helix or to a another peptide with no α-helix conformation.

###### β-peptides

Peptides predicted to have the highest amphiphilic score when adopting β-strand structure at the interface (β-20, 2.630; β-21, 3.131; β-22, 2.501; β-27, 2.213), together with peptide β-29 (score 2.027) (Table 1), led to the highest reduction in interfacial tension. However, β-24 and β-26, which also had high scores of 4.066 and 2.218 respectively, did not show the same ability to reduce interfacial tension (Fig. 2b). Moreover, β-24 was not able to stabilize the emulsion (Table 2). These findings indicated that β-24 adsorbed very poorly at the interface. This may be attributed to the short length of this peptide (7 AAs), which decrease the potential formation of an amphiphilic β-strand structure at the interface reducing the interfacial binding affinity. On the other hand, β-20, β-21 (with β-21 embedded in β-20) and β-29, which highly reduced interfacial tension, were not good emulsifiers since they led to emulsions with droplet sizes above 10 μm after production (Table 2). A plausible explanation for this may be that β-20, β-21 and β-29 are too long (≥24 AAs) resulting in peptides aggregates in the aqueous phases, which could explain the cloudy aspect of their aqueous phases (Table 2). Thus, these peptides could not stabilize the newly formed oil droplets during homogenization due to slow diffusion and slow kinetics to disintegrate the aggregates^55^.

Nonetheless, our results indicated that β-strand emulsifier peptides with middle length (13–15 AAs) such as β-22 and β-27 adopted proper re-arranging at the interface to reduce interfacial tension and also led to emulsions with the highest physical stability (e.g. droplet size <8.5 μm during storage). Interestingly, β-27 was an elongation of β-24, with six additional AAs in the C-terminal, which provided emulsifying properties to β-27 (Table 1). It should be also noted that β-22 and β-27 peptides had zero or low (≤±1.1) net charge (Table 1). This has been reported to favor adsorption of peptides at the interface (i.e. when compared to pH conditions were the AA side chains are ionized)^55^. In any case, the emulsion stabilized with β-22 had a low zeta potential (−70.9 ± 3.9 mV), which favored physical stability by causing electrostatic repulsion forces between droplets; whereas this was not the case for the emulsion stabilized with β-27 (zeta potential of 15.8 ± 2.9 mV). Similar to our results, high emulsifying activity for β-strand peptides with middle length (9–16 AAs) has previously been reported in the literature. For instance, Saito *et al*.^32^ reported high emulsifying activity for a predominantly β-strand peptide at pH 7 with 16 AAs (with alternating E and L residues). Likewise, Dexter^55^ found high emulsifying capacity for β-strand peptides with 9 AAs. It is known that using short β-strand peptides reduces the tendency of these peptides to assemble in β-sheet fibril structures in solution, increasing peptide solubility and later diffusion to the interface^55^.

Seven (β-20, β-21, β-23, β-24, β-27, β-29 and β-30) out of the eleven β-strand peptides tested in this study were not totally soluble in the buffer (Table 1), although it did not totally correlate with just length as a variable parameter. As expected, poor solubility of peptides negatively affected their emulsifying activity resulting in emulsions with low physical stability (e.g. large droplet size and phase separation) (Table 2). This may explain the poor emulsifying power of β-30 (13 AAs), is spite of its middle length; although this reasoning is not valid for β-26 (12 AAs) which was soluble. Another exception to this observed trend was peptide β-27, which could totally solubilize during homogenization allowing a fast diffusion of the peptide to the interface and final stabilization of oil droplets.

###### γ-peptides

γ-peptides with length above 18 AAs (γ-1, γ-36, γ-48 and γ-41) resulted in the lowest initial and equilibrium values of interfacial tension (Fig. 2c). It should be noted that γ-1 peptide was already identified and tested in our previous work^31^, and was included in this study for comparison. Emulsions stabilized with γ-1 and γ-36 had low D_4,3_ values after production (0.6–0.8 μm) and were physically stable during storage (e.g. no significant or slight increase of droplet size after 6 days, respectively) (Table 2). On the other hand, γ-48 was not able to totally emulsify the oil, showing an oil layer after one day storage, while emulsion stabilized with γ-41 had a large droplet size after production and phase separated soon after homogenization (Table 2). It should be also noted that other γ-peptides longer than 18 AAs (γ-38, γ-42 and γ-49) moderately decreased interfacial tension, whereas shorter peptides (<18 AAs) poorly increased surface pressure (e.g. denoting low surface activity) (Fig. 2c). For instance, γ-45, with 11 AAs and with the second highest predicted amphiphilic score (4.779), provided almost no decrease in interfacial tension (Fig. 2c). An exemption was γ-40 that with 13 AAs also reduced interfacial tension considerably (equilibrium value of ~16 mN/m). Interestingly, γ-38, γ-40 and γ-49 led to emulsions with small droplet size after production and only slight increase in droplet size during storage as well as no creaming (apart for emulsion stabilized with γ-49 at day 6), phase separation or oil layer formation (Table 2). It should be noted that γ-49 is almost totally embedded in γ-38 (except for the substitution V→I), with γ-38 having a higher γ amphiphilic score than γ-49. This is very likely due to the C-terminal DNP in γ-38 which makes the C-terminal more polar and hence the amphiphicity greater. Although emulsion stabilized with γ-42 had low droplet size after production, it presented creaming after one day of storage (Table 2).

These findings revealed that length as well as AA composition determined γ-peptides rearrangement at the oil/water interface, favoring or not the anchoring of the peptide and the shielding between oil and water molecules. Indeed, we can anticipate, as revealed from synchrotron radiation circular dichroism (SRCD), that γ-1 adopts predominant α-helix structure at the oil-water interface, whereas γ-36, γ-38 and γ-40 do not rearrange as α-helix at the interface but adopt mainly β-strand structure (γ-36) or are predominantly unordereded (γ-38 and γ-40) (manuscript in preparation). Moreover, it is remarkable that, besides the different net charge values observed for γ-1, γ-36, γ-38, γ-40, γ-49 (ranging from −1 to 5 at pH 7), physically stable emulsions stabilized with these peptides in general had large absolute zeta potential values (>30 mV). An exception to this was emulsion stabilized with γ-49 which could explain the creaming observed for this emulsion at day six of storage. Conversely, emulsions which were not physically stable (e.g. those stabilized with γ-34, γ-35, γ-39, γ-45, γ-46 γ-47) presented a low absolute zeta potential value (<30 mV) (Table 2), in spite of having net charge varying from -0.1 to 5 at pH 7 (Table 1). This may be explained by a low amount of these peptides adsorbing at the interface, as a consequence of a low emulsifying activity. In addition, the results indicated that net charge of γ-peptides did not control adsorption of the peptides at the oil/water interface, which seemed to be mainly influenced by peptide re-arranging to enable its hydrophobic patches to bind the oil phase at the interface^56^. Thus, the larger mainly hydrophobic regions of γ-1 and γ-36 peptides (14 and 17 AAs, respectively) when compared to γ-38, γ-40 and γ-49 (8 AAs) (Table 1) may have favored the interaction of the former peptides with the oil at the interface. It correlated well with the higher reduction of interfacial tension observed for γ-1 and γ-36 when compared to γ-38, γ-40 and γ-49, and with the higher physical stability of emulsions stabilized with γ-1 and γ-36 (especially the one stabilized with γ-1, which showed the lowest droplet size and no significant change during storage as well as no creaming) (Table 2). It is also worth mentioning that γ-1 (28 AAs) showed lower initial and equilibrium interfacial tension values when compared to sodium caseinate (Fig. 2c) and emulsion stabilized with γ-1 had higher physical stability than the one stabilized with sodium caseinate (Table 2). This is in agreement with our previous study^31^, and correlated well with its high γ amphiphilic score obtained when using our improved prediction algorithm (4.146) (Table 1).

Peptide γ-39, which was a shorter version of γ-38 and γ-49 and had the highest predicted amphiphilic score (4.790), 11 AAs and a short mainly hydrophobic region (4 AAs) (Table 1), reduced interfacial tension but with a considerable long lag time to reach equilibrium value (Fig. 2c). Moreover, emulsion stabilized with γ-39 phase separated right after homogenization (Table 2), denoting the poor emulsifying activity of this peptide. γ-39 may be too short to lead to an amphiphilic α-helix. Furthermore, γ-39 was soluble (Table 2), indicating low or no formation of β-sheet fibril structures in solution which could hinder its diffusion to the interface. Therefore, a more plausible explanation for the long lag time observed to reduce interfacial tension for γ-39 could be a perpendicular adsorption at the interface. Perpendicular adsorption and packing of peptides at the interface, specially the peptides evaluated in this study above 7 AAs, will likely be much slower when compared to axial adsorption. As a consequence, γ-peptides found to lead to low initial and equilibrium values of interfacial tension as well as to exhibit high emulsifying activity (γ-1, γ-36, γ-38, γ-40 and γ-49) may orientate parallel to the interface and in a well-defined secondary structure rather than perpendicularly. Parallel orientation might maximize inter-peptide interactions strengthening the interfacial layer^30^, which could enhance physical stability of the peptide-stabilized emulsions.

Among the 38 peptides tested, α-10, α-12, β-22, β-27, γ-1, γ-36, γ-38, γ-40 and γ-49 showed the best emulsifying properties. Thus, these nine emulsifier peptides were selected for further studying, including peptide structure determination, antioxidant activity evaluation and release from potato side-streams.

##### Protein modelling for selected peptides: Localization and potential structure determination

The local structure within a native protein can provide insight into a potential structure of an isolated peptide. For instance, Poon *et al*. (1999) isolated a surface exposed, predominantly α-helical peptide from horse myoglobin, that displayed a more rapid decrease of surface tension and smaller droplet sizes than the intact protein, indicating that the amphiphilic character of the peptides was retained at the interface. Predicted emulsifier peptides will likely be found in surface exposed regions as these regions will (at least to some extent) display an amphiphilic behavior in aqueous solution. Consequently, the native conformation within the surface exposed region of the protein could indicate a potential structure at the oil/water interface. Using templated homology modelling, we identified the localization and probable structure of the nine selected emulsifier peptides within their protein of origin (Fig. 3).

For the four PAT2B peptides (α-10, α-12, γ-1, and γ-36), two were localized in the same region (α-10 and α-12) with predominant α-helical conformation (α-12 fully embedded in α-10, as previously mentioned). γ-1 was also localized in a predominantly α-helical region of PAT2B, whereas γ-36 was localized in a region that comprises both α-helical and β-strand content as well as almost 50% unordered structure. This correlates well with the aforementioned SRCD data (manuscript in preparation), where α-10, α-12, and γ-1 all adopt a mainly α-helical structure at the interface. The superior emulsifying activity of γ-1 may be ascribed to formation of a longer and more amphiphilic helix than α-12 and α-10, respectively, in agreement with our model (Fig. 3 and Table 1).

In KTI-A, β-27 contains an N-terminal β-strand domain and a small C-terminal α-helical domain connected by a flexible region comprising roughly half of the peptide. The C-terminal cysteine has been reported to be key in homo-dimer formation^41^. With the loss of the disulfide restriction and the large variable domain, the interfacial structure of isolated β-27 may differ from that found in our KTI-A model. Nevertheless, the strand domain of β-27 consists of alternating polar/non-polar residues characteristic of a good β-emulsifier, which could explain the observed activity.

Remarkably, all four KTI-B peptides (β-22, γ-38, γ-40, and γ-49) were localized in the same 31 residue region of the protein (C182-P212), although γ-49 contains a single V->I substitution, and hence must be found in a different KTI-B isoform than β-22, γ-38, and γ-40. The region includes two antiparallel β-strands (almost 50%) while the rest is predominantly unordered. In this respect, it should be mentioned that the N-terminal part of both β-22 and γ-40 was localized in a very flexible region of the Q3S474 KTI-B model (P175-F190) that has a low local quality estimate (QMEAN <0.75) (Fig. 1S). Consequently, the structure of this region was hard to predict by templated modelling. γ-38 and γ-49 (with γ-49 fully embedded in γ-38 when disregarding the point mutation, as previously described) both contain the antiparallel β-sheet, but as this is not intrinsically amphiphilic (both peptides are predicted as γ emulsifiers), the specific structure at the interface is also hard to predict. It should be noted, that β-22, γ-38, and γ-40 can also be found in e.g. Q2XPY0, which does not contain the flexible region upstream of the peptides, but the anti-parallel sheet structure is retained. Interestingly, SRCD data indicate that only β-22 adopted a predominantly β-strand conformation at the interface (manuscript in preparation). Although γ-38 and γ-49 contain the antiparallel β-sheet, this could indicate that this specific conformation is largely governed by forces on the global protein level rather than by local forces and propensity for β-strand of the individual AAs. The more defined secondary structure of β-22 could also explain the lower surface tension for this peptide compared to the three γ-peptides from the same region. The fraction (%) of AAs found in a well-defined secondary structural element (i.e. α-helix or β-strand) for each of the selected peptides is summarized in Table 1.

#### Antioxidant activity of the selected emulsifier peptides

Peptide emulsifiers exhibiting antioxidant activity are preferred since they can also provide chemical stability to the peptide-stabilized emulsion (e.g. oxidative stability). This is of special relevance for emulsions containing omega-3 polyunsaturated fatty acids, which are common delivery systems used for the enrichment of food matrices with these healthy, but highly prone to oxidation, lipids^25,26^.

**Figure 4:**
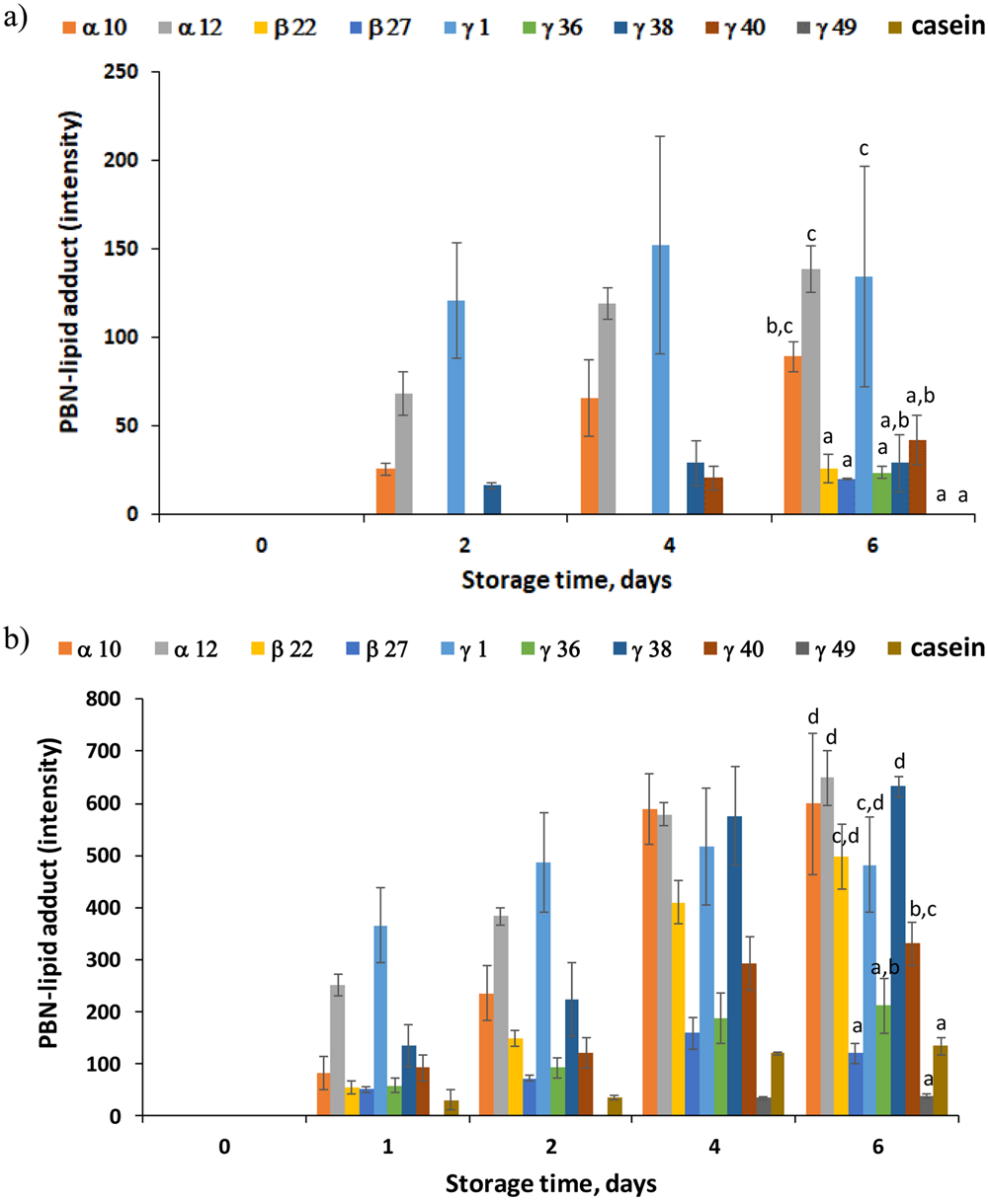
PBN-lipid derived spin adducts generation resulting from the oxidation of 5 wt.% fish oil-in-water emulsions stabilized with selected synthetic potato peptides during storage in the dark at: (**a**) 20 °C, and (**b)** 37 °C. For the last sampling point, different letters indicate significant differences between samples (p < 0.05).

Figure 4 shows the generation of PBN-lipid spin adducts during storage of fish oil-in-water emulsions at 20 °C (Fig. 4a) and 37 °C (Fig. 4b). PBN-lipid spin adducts, which are stable radicals and thus measurable by ESR, were formed by reaction between PBN and highly reactive lipid radicals (i.e. peroxyl and alcoxyl radicals)^57^. Moreover, similar ESR spectra were obtained for PBN-adducts in emulsions and in bulk fish oil (i.e. with the common three broad lines characteristics for the coupling for nitroxyl radicals), indicating that the radicals trapped by PBN were produced in the oil phase of the emulsions^31,58^. Hence, the generation of PBN spin adducts in peptide-stabilized emulsions gives information about the oxidation status of the emulsions, and thus the chemical stability provided by the peptides^59^. As expected, lipid oxidation was accelerated with temperature, resulting in higher generation of PBN-spin adducts for emulsions stored at 37 °C than at 20 °C.

**Figure 5:**
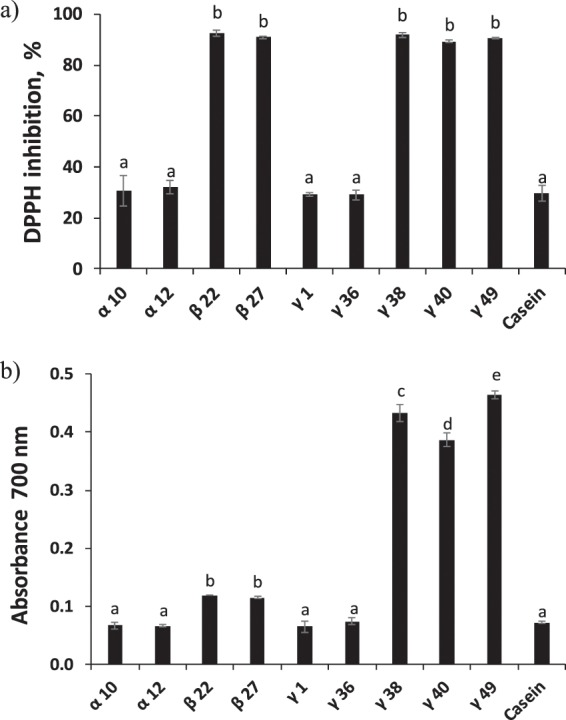
*In vitro* antioxidant capacity of selected synthetic potato peptides (0.2 wt.% at pH 7): (**a**) DPPH radical scavenging activity, and (**b**) reducing power. Different letters indicate significant differences between samples (p<0.05).

It was observed that emulsions stabilized with α-10, α-12, and γ-1 showed the lowest oxidative stability (i.e. highest generation of PBN-lipid spin adducts) at both temperature values assayed (Fig. 4). Furthermore, a light brownish color was observed for emulsions stabilized with α-12 and γ-1 after four and one day of storage at 37 °C, respectively (data not shown). In our previous study^31^, we also observed an intense brown color for a fish oil-in-water emulsion stabilized with γ-1 when stored at 50 °C, which was associated with advanced oxidation (i.e. protein co-oxidation including non-enzymatic browning reactions). Interestingly, although emulsions stabilized with β-22 and γ-38 were significantly more oxidatively stable at 20 °C compared to emulsions stabilized with α-10, α-12, and γ-1 (Fig. 4a), they did not show significant differences when stored at 37 °C (Fig. 4b). The superior oxidative stability of emulsions stabilized with β-22 and γ-38 when stored at 20 °C can be attributed to the significantly higher DPPH scavenging activity of these peptides compared to α-10, α-12, and γ-1 (Fig. 5a); with γ-38 also showing significantly higher reducing power (Fig. 5b). Contrary to α-10, α-12, and γ-1, β-22 and γ-38 present one C residue in their sequence (Table 1). C is a highly oxidizable AA, which easily donates the proton from the SH group enhancing free radical scavenging^60^. Moreover, β-22 and γ-38, as well as γ-1 but not α-10, α-12, present one F residue (Table 1), which favors radical scavenging by direct electrons donation^61^.

Emulsions with the highest oxidative stability, both at 20 and 37 °C, were those stabilized with β-27, γ-36, γ-40, γ-49 and sodium caseinate (Fig. 4). β-27, γ-40 and γ-49 presented higher DPPH radical scavenging than γ-36 (Fig. 5a), since they present one C and one F residues which favor protons and electrons donation, respectively (Table 1). Interestingly, the sequence FCLKVGV was embedded in all the peptides showing significantly higher DPPH radical scavenging activity than the other peptides, apart from β-27 (Table 1). This sequence, containing F, C and K residues with reported radical scavenging properties^61^, as well as hydrophobic AAs (L, V and G) which facilitates the interaction between peptides and lipid radicals, may be responsible for the radical scavenging activity observed for β-22, γ-38, γ-40 and γ-49. It should be also noted that for the nine selected emulsifier peptides, negligible Fe^2+^ chelating activity was found (ranging from 0 to 7.3%) when compared to sodium caseinate (98.5 ± 0.2%) (data not shown). These peptides, apart from β-27, contained AA residues with carboxyl groups at the side chain such as E and D, and also H residue, which shows chelating activity through its imidazole group (only in γ-38 and γ-49). Nonetheless, the lack of chelating activity could be attributed to an excessive length of the peptides when compared to common chelator peptides (e.g. <1 kDa), leading to a low negative charge-to mass ratio^18^.

In any case, oxidative stability of emulsions cannot be totally explained by antioxidant activity of emulsifier peptides, as denoted by the low oxidative stability of emulsion stabilized with γ-38 and β-22 at 37 °C (Fig. 4b). For instance, physical destabilization during storage, which degrades protein film at the interface, may favor the contact between prooxidants and the emulsified oil^26^. Nevertheless, this is not a reasonable explanation for the emulsion stabilized with γ-1, with the lowest oxidative stability (Fig. 4) and constant droplet size during storage both at 20 and 37 °C (Fig. 2S); whereas for instance γ-36 emulsion destabilized during storage at both temperatures (Fig. 2S) and presented a low formation of PBN-lipid spin adducts (Fig. 4). Furthermore, caution should be taken when discussing results at 37 °C for emulsions stabilized with β-22, β-27, γ-49 and caseinate, since they presented a creamed layer after day one. Moreover, although these emulsions recovered their normal physical structure after vortexing, this was not the case for emulsions stabilized with β-27, which formed white particles in the aqueous phase after applying vortex. A complementary study on oxidative stability of emulsions stabilized with the selected nine emulsifier peptides, measuring primary oxidation products (e.g. peroxides) and specific secondary volatile oxidation products, is on-going in our lab.

### Proteomics analysis of potato processing side streams

#### Abundant proteins in potato processing side streams

In conventional bottom-up proteomics studies, quantification is performed using exclusively unique peptides in order to distinguish between proteins and protein isoforms with certainty. Consequently, razor peptides would be omitted from quantitative analysis, as inclusion may dramatically skew the actual protein-to-protein ratio. However, as we in this study aim to quantify the total content of certain protein classes, omitting razor peptides would produce a significant negative bias towards protein classes with high degrees of homology. This implies that e.g. patatin content would be significantly underestimated, as patatins include many consensus regions and regions with very high homology between the different isoforms. Furthermore, inclusion of razor peptides also implies that the relative abundance of specific patatin isoform quantified in this work needs to be used as indicative, as inclusion of said razor peptides will introduce a bias towards certain isoforms based of the identified unique peptides and the combinatorial statistics applied in the underlying quantitative algorithm.

**Figure 6:**
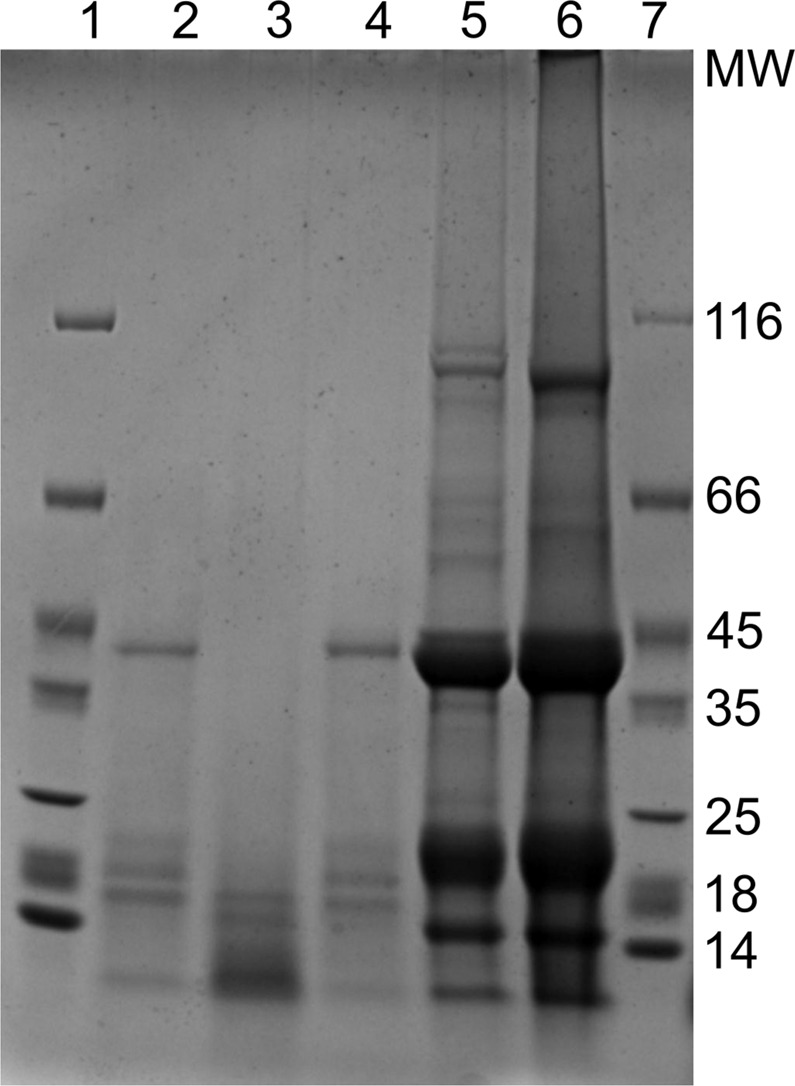
SDS-PAGE (4–20% gradient gel) analysis of the potato side stream samples analyzed in this work. 1: MW marker (Pierce Unstained Protein MW Marker P/N 26610). 2: AKV-Feed. 3: AKV-K2. 4: KMC-Feed. 5: KMC-Food. 6: AKV-PFJ. 7: MW marker.

Even though all side stream samples were prepared to the same apparent protein concentration (2 mg/mL based on provided Kjeldahl-N), there are clear differences in both protein content and, naturally, distribution. From SDS-PAGE analysis (Fig. 6), it was evident that with increasing protein processing, the concentration of (soluble) protein decreased significantly. The protein content in raw PFJ and non-denatured extract (lanes 5 and 4, respectively) was significantly higher than what was observed for heat/acid extracted protein (lanes 1 and 3), and even more so than for the protamylasse fraction (lane 2). This observation was indeed also reflected in the LC-MS/MS data (Table 3S), where similar differences were found for the number of identified peptides and protein groups between the tryptic digests.

Identified proteins were quantified using the iBAQ algorithm to determine the relative protein distribution within each sample, and all proteins with riBAQ> 1% on the sample level were selected for further bioinformatics processing. No additional peptides with a significantly higher score than those already predicted from the initially selected patatins and KTI proteins, were identified. This verified the good coverage of abundant potato proteins within the initial selection. The list of abundant proteins (Tables 4S–6S) consisted primarily of different isoforms of patatin, KTIs, proteinase inhibitors (PI/PINs), and metallocarboxypeptidase inhibitors (MCPIs). Furthermore, the list included various cold-stress related proteins, heat shock proteins, lipoxygenases (LOXs), histones, ripening-related proteins, RNA-binding proteins, translation initiation factors, and pathogenesis-related proteins.

**Figure 7:**
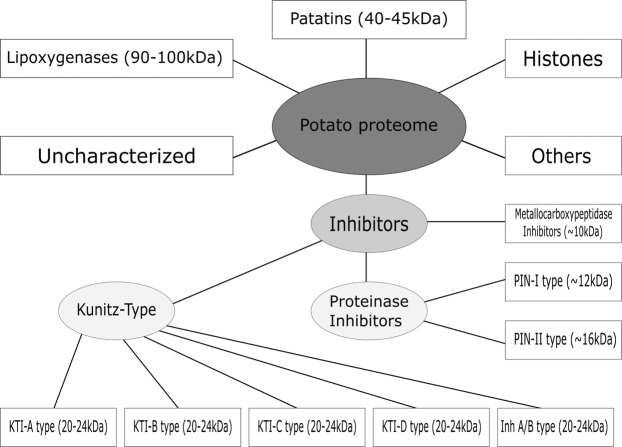
Overview of protein structural/functional classes (i.e. proteins with a high degree of sequence conservation) within the potato proteome with emphasis on the most abundant classes based on riBAQ abundance determined in this work. End groups (illustrated in squares) are regarded as sufficiently related to be regarded as isoforms of the same proteins with a high probability of localizing identical embedded peptides. The figure was created using INKSCAPE version 0.92.3 (https://inkscape.org/).

Proteins were subsequently grouped according to sequence identity in order to produce classes of highly homologue proteins from which it will be possible to identify identical or highly related peptides. Doing so, it is also possible to estimate a potential yield in the isolation of a given peptide from a complex sample under optimal conditions. Traditionally, potato proteins have been grouped into fractions of patatins, LOXs, and protease inhibitors, where the latter is often divided into subgroups of KTIs, PINs and MCPIs^14,15,24,62–66^. Nevertheless, this division proved insufficient to produce groups of satisfactory sequence homology. Consequently, we created a class model, where especially KTIs and PINs are divided into underlying subgroups (Fig. 7). Based on our results of abundant proteins, this model was sufficient to describe the diversity of potato proteins in terms of sequence homology.

In order to compare our quantitative findings with existing literature, subgroups were collapsed and regarded as one general protein class (Table 3). In this respect, it should be mentioned that 1D SDS-PAGE is a very simple and non-specific method, and undesired proteins may be co-determined in any given band thereby potentially overestimating the content of a specific protein group. Overall, we found quite good correlation between our PFJ analysis and that of Bauw *et al*.^14^. Our MS-based results were slightly underrepresented in high MW proteins (LOXs) and overrepresented in low MW proteins (all classes of inhibitors). This was a direct result of molar (MS-based) and weight (SDS-PAGE) distributions not being directly comparable and such a bias could be expected. Furthermore, upwards of ¼ of the identified proteins fall into the “uncharacterized” and “other” categories. Looking at the different side streams, there were significant differences compared to the PFJ. In KMC-Food, we observed a comparable protein distribution making it a representative protein isolate for the unprocessed PFJ. In the heat/acid precipitated samples (KMC-Feed and AKV-Feed), the patatin fraction was significantly enriched (relatively), while the inhibitors and especially the KTIs and MCPIs were somewhat depleted. This result is as expected, as patatins are generally regarded as acid-coagulable whereas many inhibitors are regarded stable towards acid and heat due to their high degree of (conserved) disulfide bonding^62^. Consequently, the low MW fraction was highly enriched in the protamylasse fraction (AKV-K2), as this can be regarded as the eluate after protein precipitation. MS-based relative quantification (Table 3) did not reflect this clear result, which was likely due to AKV-K2 also containing significant amounts of partially hydrolyzed protein from the acid-coagulable fraction, seen as the low MW blur on SDS-PAGE (Fig. 6). Consequently, finding a quite high relative abundance of patatins (27%) does not indicate that the fraction contains a significant amount of intact patatin, but rather unspecific fragments from acid-induced hydrolysis occurring during precipitation.

**Table 3:** Relative molar distribution of quantified potato proteins by MaxQuant riBAQ analysis of LC-MS/MS data and apparent weight distribution based on 1D SDS-PAGE lane intensity profile analysis according to classes outlined in Fig. 7.

Protein Class	Molecular weight	PFJ* (ref)	AKV-Feed		AKV-K2		KMC-Feed		KMC-Food		AKV-PFJ	
			MS	SDS**	MS	SDS**	MS	SDS**	MS	SDS**	MS	SDS**
Lipoxygenase	90–100 kDa	~10%	0.8%	—	0.2%	—	0.8%	—	1.4%	3.9%	3.2%	6.0%
Patatin	40–50 kDa	25–40%	56%	25%	27%	—	60%	28%	29%	35%	28%	29%
Inhibitors	—	~40%	31%	68%	33%	100%	23%	72%	53%	58%	44%	62%
− KTIs	20–24 kDa	~30%	30%	48%	15%	12%	22%	43%	51%	42%	41%	37%
− PINs	12–16 kDa	~5%	1.2%	12%	13%	33%	0.5%	14%	1.7%	15%	2.8%	16%
− MCPIs	~10 kDa	~4%	0.2%	8.4%	4.8%	55%	0%	15%	0%	1.7%	0.1%	8.0%
Histones	N/A	~1%	0.8%	N/A	3%	N/A	1.2%	N/A	0.1%	N/A	0.7%	N/A
Uncharacterized	N/A	N/A	4.7%	N/A	18%	N/A	6.3%	N/A	8%	N/A	11%	N/A
Other	N/A	N/A	7%	N/A	19%	N/A	9%	N/A	9%	N/A	13%	N/A

All Kunitz-type inhibitors and proteinase inhibitors have been grouped for simplicity. *PFJ protein distribution (by weight) as determined by UV/Vis spectrophotometry and BCA assays based on Bauw *et al*. (2006).

**SDS-PAGE-based distribution is based only on MW-range distribution and may include other proteins of comparable MW.

In the light of the above, it would be of high interest to target patatin-derived emulsifier peptides from the heat/acid precipitated protein extracts (AKV-Feed and KMC-Feed), whereas KTI-derived peptide emulsifiers would have the highest potential yield in the non-denatured protein extract (KMC-Food) and more interestingly, the unprocessed PFJ.

#### Abundance of selected peptides in potato side streams and potential release by tryptic hydrolysis

For the nine selected peptides displaying promising emulsifying activity, we employed MSA using all identified proteins within the (sub)group related to the peptide in order to identify protein isoforms with both 100% and> 90% identity to the predicted peptide. Consequently, we can estimate the potential yield in release of both predicted and highly homologue peptides (in relative molar yield) based on quantitative protein-level data (Table 4). It was observed that all patatin-derived peptides (α-10, α-12, γ-1, and γ-36) and their related homologues were significantly enriched in the heat/acid-precipitated protein extracts, and in over two-fold higher abundance in the KMC-Feed protein extract, while the abundance was comparable to AKV-PFJ in both AKV-K2 and KMC-Food. This is in agreement with the proteomics analysis revealing that patatins appear to be enriched in heat/acid precipitated potato protein extract (Table 3). Allowing for small sequence variations (>90% identity), all patatin-derived peptides were in fact found in more than half of the identified proteins in both heat/acid precipitated protein extracts (AKV-Feed and KMC-Feed) indicating a potentially very high yield from these side streams. This is of high industrial relevance, as this is the commonly used method for protein extraction in the potato processing industry due to low cost and high yield.

**Table 4:** Sequence identity between quantified protein isoforms (patatins, Kunitz type-A, and Kunitz type-B) and the selected nine peptides from bioinformatic prediction and *in vitro* functional validation.

Sample	Peptide	Patatin				KTI-A	KTI-B			
	Identity	γ-1	γ-36	α-10	α-12	β-27	β-22	γ-38	γ-40	γ-49
AKV-Feed	100%	20.2%	50.3%	26.2%	26.2%	8.8%	5.1%	5.1%	5.1%	0.0%
	>90%	50.5%	54.5%	50.5%	50.5%	11.3%	5.1%	5.1%	7.6%	5.1%
AKV-K2	100%	9.5%	24.5%	14.5%	14.5%	4.1%	5.5%	5.5%	5.5%	0.0%
	>90%	25.2%	25.9%	24.5%	24.5%	5.8%	5.5%	5.5%	7.0%	5.5%
KMC-Feed	100%	43.6%	53.8%	37.1%	37.1%	8.3%	3.4%	3.4%	3.4%	0.0%
	>90%	54.5%	59.0%	54.1%	54.1%	10.2%	3.4%	3.4%	3.4%	3.4%
KMC-Food	100%	11.1%	26.0%	13.8%	13.8%	12.3%	6.9%	6.8%	6.9%	0.1%
	>90%	17.0%	28.9%	26.1%	26.1%	15.9%	6.9%	7.0%	10.2%	7.0%
AKV-PFJ	100%	16.9%	21.7%	16.3%	16.3%	8.9%	6.2%	6.2%	6.2%	0.2%
	>90%	24.3%	26.8%	22.0%	22.0%	12.9%	6.2%	6.5%	8.4%	6.5%

Relative abundance (in % riBAQ within each sample) is given for all proteins with 100% identity and >90% identity with the respective predicted peptide. Sequence identity evaluation was performed using CLC SEQUENCE VIEWER version 8.0 (https://www.qiagenbioinformatics.com/).

The KTI-A-derived β-27 was found in approximately 10% of the identified proteins in AKV-PFJ, AKV-Feed, and KMC-Feed, while the abundance was around 50% higher and lower in KMC-Food and AKV-K2, respectively. Although generally enriched in the low MW region, intact KTIs (20–24 kDa) appeared depleted in AKV-K2 based on the SDS-PAGE analysis (Fig. 6), thereby explaining the low abundance of KTI-A-and the derived β-27. For the KTI-B-derived peptides, the abundance was estimated to be 3–7% for β-22, γ-38, and γ-40 in the analyzed samples with highest abundance in KMC-Food and AKV-PFJ, and thereby still in a reasonable level to target for release. KTI-B isoforms containing γ-49 were only identified at extremely low abundance in KMC-Food and AKV-PFJ thereby making the pursuit hereof highly unfavorable.

Using the peptide-level data from the proteomics analysis, it is possible to determine if the predicted peptide emulsifiers are actually experimentally identified and, moreover, to estimate the potential for isolating by means of tryptic hydrolysis. Based on the identified proteins showing 100% identity in the regions containing the selected peptides, we performed peptide-level MSA using all identified tryptic peptides to investigate the experimental sequence coverage across samples and predicted peptides (Table 7S). Interestingly, there was high (>75%) or full experimental sequence coverage for γ-1, which was the most potent emulsifier, in all samples. This verified that the patatins, from where γ-1 can be released, were in fact intact in the region of interest. Especially interesting is this observation for KMC-Feed, where the γ-1 region had full sequence coverage in 42.5% of all identified protein (Table 7S) with the peptide being identified in 43.6% of proteins in total (Table 4). Furthermore, this implies that for each mol of protein processed, (at least) 425 mmol of γ-1 can potentially be released. Similarly, for γ-36, full coverage was observed in almost all identified protein containing the region. An exception to this was AKV-K2, where no experimental coverage was observed further substantiating our claim that this side stream predominantly contains fragments of patatins. The experimental coverage for α-10 and α-12 was below 75% in all samples. It is also noteworthy that the β-27 region in KTI-A appeared intact in all samples, as full experimental sequence coverage was observed. The experimental sequence coverage for KTI-B derived peptides (disregarding γ-49) was quite comparable and, with the exception of AKV-K2, in the partial to full range. This is not surprising as the three peptides were located in the same region of KTI-B.

Incomplete sequence coverage may be a direct consequence of the sequence of the predicted peptides and the surrounding regions, as tryptic hydrolysis may produce either too short or too long peptide fragments incompatible with a bottom-up proteomics approach. This will also have a direct impact on the potential of releasing the peptides by means of tryptic hydrolysis. In order to determine this, the sequences of the nine selected peptides was outlined (Table 5), including a 15 residue N- and C-terminal cleavage window based on the protein sequences of the modelled proteins (Fig. 4). Besides the high abundance of γ-1 and γ-36, the positioning of the tryptic residues (i.e. R or K) are favorable to achieve the high to full sequence coverage observed experimentally. Interestingly, the placement of the tryptic residues in both peptides also favors tryptic release of almost full length peptides. γ-1has a lysine in position 3 (K3) as well as an arginine in the last position (R28). The fact that we also find a tryptic residue in K25 may not be detrimental, as it is followed by an acidic residue in D26. Acidic (and generally charged residues) found in the P1’ position of a tryptic cleavage site is known to decrease the rate of hydrolysis substantially^67^, thereby favoring cleavage after R28. This observation was also verified (based on raw MS1 peptide intensity) as we observed at least 10-fold higher abundance of the longer variant including the C-terminal DAR compared to the C-terminally truncated variant (i.e. without DAR) (data not shown). Consequently, the release of γ-1 with a three AA truncation at the N-terminus is highly feasible using trypsin.

**Table 5:** Sequences of the nine selected emulsifier peptides including a 15 AA N- and C-terminal cleavage window.

Peptide	Protein	Sequence (w. cleavage windows)
γ-1	P15477	A**K**LEEMVTVLSIDGG *GI****K****GIIPAIILEFLEGQLQEVDNN****K****DA****R*** LADYFDVIGGTSTGG
γ-36	P15477	LQEVDNN**K**DA**R**LADY *FDVIGGTSTGGLLTAMITTPNENN****R****P* FAAA**K**DIVPFYFEHG
α-10	P15477	EANMELLVQVGETLL ***KK****PVS****K****DSPETYEEAL****KR****FA****K****LLSD****RKK****L* **R**AN**K**ASH*
α-12	P15477	LVQVGETLL**KK**PV**SK** *DSPETYEEAL****KR****FA****K****LLSD* **RKKLR**AN**K**ASH*
β-27	Q3S488	V**R**FIPLSTNIFEDQL *LNIQFNIPTP****K****LC* VSYTIWKVGNINAPL
β-22	Q3S474	QLGYNLLYCPVTSTMI *CPFSSDDQFCL****K****VGV* VHQNG**KRR**LALV**K**DN
γ-38	Q3S474	PVTSTMICPFSSDDQ *FCL****K****VGVVHQNG****KRR****LALV****K****DNP* LDVSF**K**QVQ*
γ-40	Q3S474	NLLYCPVTSTMICPF *SSDDQFCL****K****VGVV* HQNG**KRR**LALV**K**DNP
γ-49	Q3S477	YCPATMICPFCSDDE *FCL****K****VGVIHQNG****KRR****LALV****K*** DNPLDVSF**K**QVQ*

The sequence of the predicted peptide is highlighted in italics and tryptic residues are highlighted in bold. Several peptides are located close (<15 AAs) to the protein C-terminus and * indicates the terminal residue.

γ-36 has an arginine located 5 residues upstream of the peptide thereby introducing an LADY elongation to the N-terminus. An arginine is also found at the second to last residue (R25), but since it is followed by proline, which is known to potentially impair cleavage if located in position P1’^68^, the peptide may cleaved at the lysine 5 residues downstream, thereby introducing a C-terminal FAAAK elongation. This peptide was experimentally identified in all samples (with exception of AKV-K2), while the shorter form cleaved before P was only identified in AKV-PFJ at a very low abundance.

For α-10 and α-12, (α-12 fully embedded in α-10) the placement of tryptic residues are unfavorable in terms of potential tryptic release, as they contain nine and three tryptic residues, respectively. The high amount of tryptic residues and their close proximity makes a large proportion of the released tryptic peptides too short for identification using classical bottom-up proteomics, where peptides are preferably above six residues to decrease the number of potential proteins of origin during peptide mapping. This is also the basis for the significantly lower sequence coverage compared to the patatin-derived γ-peptides. Consequently, the most representative tryptic peptide is DSPETYEEALK(R). Interestingly, this peptide, especially the variant including the C-terminal arginine, is found in extremely high abundance based on a relative MS1 intensity.

In the KTI-A-derived β-27, a lysine is located at the third to last position. However, no tryptic residues are found in the N-terminal region, and tryptic hydrolysis would introduce a 13 residue N-terminal elongation thereby doubling the peptide length, which presumably will have a large impact on peptide function. Nevertheless, combining tryptic hydrolysis with e.g. Asp-N Endopeptidase, which would cleave on the N-terminal side of the aspartic acid^69^ three residues upstream of β-27, thereby releasing a peptide very closely resembling β-27, could be feasible.

As previously descripted, all three Q3S474-derived KTI-B peptides (β-22, γ-38, and γ-40) are located within the same 31 residue region, and consequently overlap with one another. Unfortunately, the shared core peptide (FCLKVGV) has a centrally placed lysine which would facilitate hydrolysis thereby removing this region, which may be directly responsible for radical scavenging, as previously described. In addition to this, the preceding tryptic residue is a lysine located 30 residues upstream from the central lysine (i.e. outside the N-terminal cleavage window in Table 7S), thereby producing a very long N-terminal extension to any tryptic peptide. The addition of at least 18 residues to the N-terminal could make the function of the variant peptides dramatically. Likewise, for γ-49 (which also includes the shared FCLKVGV core peptide), the preceding lysine is located 28 residues upstream. It should be noted that if the Q3S474-derived peptides are isolated from Q2XPY0 (which is missing an 8-redisue stretch in the upstream region (VTSTSMICP)), the peptides would have a significantly shorter extension. Although this variant (SSQLGYNLLYCPFSSDDQFCLK) was experimentally identified, it will likely still have a significantly different activity. As the region with all four KTI-B peptides also contains multiple tryptic residues (including a KRR stretch), the number of potential tryptic peptides becomes large. And since the majority of these peptides are not identified, and the identified ones are only present in relatively low abundance, the release of any of these peptide by tryptic hydrolysis seems unfeasible.

Until now, we have only looked at tryptic peptides that have 100% identity with (parts of) the predicted peptide emulsifiers. However, as potatoes contain several isoform of the most abundant proteins (i.e. patatins and protease inhibitors), the potential yield will increase significantly if small differences are allowed in the tryptic peptides (Table 4). Whether the isoform variants and truncated/elongated tryptic variants of the selected peptides discussed above will still exhibit the same impressive emulsifying activity, is under investigation in our laboratories.

## Conclusions

In this study, we demonstrated the potential of a novel combination of bioinformatics prediction, functionality testing and bottom-up proteomics to identify peptide emulsifiers embedded in potato proteins. The results indicated that although the amphiphilic vector of a peptide in a given conformation did not fully predict the emulsifying activity of peptides, it provided a decent preliminary estimate of their emulsifying potential (i.e. there is not necessarily activity for a high score but there is a good probability of functionality). Peptides above 18 amino acids, which were predicted to be emulsifiers having α-helix structure, showed more potential to adopt amphiphilic α-helix conformation at the interface when compared to shorter peptides (<14 AAs). On the other hand, β-strand peptides with middle length (13–15 AAs), which reduces the tendency of these peptides to assemble in β-sheet fibril structures in solution, had higher emulsifying activity when compared to longer peptides. γ-peptides preferentially adsorbed parallel to the interface (e.g. in α-helix or β-strand conformation) rather than perpendicularly, where the existence of long hydrophobic regions favored the anchoring at the interface.

Furthermore, using quantitative proteomics, we were able to estimate the molar abundance of the predicted peptide emulsifiers and their closely related isoforms in different side streams from the potato processing industry. The most potent peptide emulsifier, γ-1, was identified in over 40% of the quantified protein in a feed-grade protein extract obtained by conventional heat/acid precipitation due to its patatin enrichment and in almost 20% of the protein from the unprocessed potato fruit juice. Similar abundances was seen for the other patatin-derived peptide emulsifiers. Allowing for small variations in the sequences and including the peptide isoforms, the potential yield is even greater. Peptide-level proteomics data and sequence analysis revealed that it would be possible to obtain γ-1 in almost full length by means of simple tryptic hydrolysis. Potent peptide emulsifiers derived from different Kunitz-type protease inhibitors were also identified and, in contrast to patatin-derived peptide emulsifiers, displayed significant oxidative stability. The majority was found in around 10% of the protein in the raw PFJ. The most abundant of these, β-27, would be possible to obtain in almost intact form by using sequential/combinatorial hydrolysis with trypsin and Asp-N.

## Supplementary information

Supplementary information.

## Data Availability

The datasets generated during the current study are available on reasonable request.
