# Supplementary material for: Identification of emulsifier potato peptides by bioinformatics: application to omega-3 delivery emulsions and release from potato industry side streams
Source: Sci Rep. 2020 Jan 20;10:690. doi: 10.1038/s41598-019-57229-6 (PMC6971092; doi:10.1038/s41598-019-57229-6)
Supplement: Supplementary file 1 — Supplementary information. [file 41598_2019_57229_MOESM1_ESM.pdf]

SUPPLEMENTARY MATERIAL

Identification of emulsifier potato peptides by bioinformatics: application to omega-3 delivery emulsions and release from potato industry side streams

Pedro J. García-Moreno[1*], Simon Gregersen[2], Elham R. Nedamani[1], Tobias H. Olsen[3], Paolo Marcatili[3], Michael T. Overgaard[2], Mogens L. Andersen[4], Egon B. Hansen[1], Charlotte Jacobsen[1]

[1] National Food Institute, Technical University of Denmark, Denmark

[2] Department of Chemistry and Bioscience, Aalborg University, Denmark

[3] Department of Bio and Health Informatics, Technical University of Denmark, Denmark

[4] Department of Food Science, University of Copenhagen, Denmark

Pedro J. García-Moreno and Simon Gregersen contributed equally to this work.

[*] Corresponding author: pejeg@food.dtu.dk

Table 1S. Initially selected proteins from *Solanum tuberosum* used to identify emulsifier peptides by bionformatics

Entry	Protein	Length
P15478	Patatin-T5	386
P15477	Patatin-B2	386
Q3YJT5	Patatin-05	387
P11768	Patatin group M-1	386
Q2MY60	Patatin-02	387
Q2MY43	Patatin-08	387
Q42502	Patatin-2-Kuras 3	386
Q3YJS9	Probable inactive patatin-3-Kuras 1	374
Q3YJT3	Patatin-2-Kuras 1	374
Q8RXA3	Cathepsin D inhibitor (KTI-A protein) (Kunitz-type enzyme inhibitor P4E1)	220
Q3S474	KTI-B protein (Kunitz-type protease inhibitor)	221
Q3S477	KTI-B protein (Kunitz-type protease inhibitor)	219
Q2XPX8	KTI-B protein (Kunitz-type protease inhibitor-like protein)	213
Q3S488	Kunitz-type protease inhibitor	220
Q3S481	Kunitz-type protease inhibitor	220
A0A097H149	Kunitz-type inhibitor B	221
Q38M77	Kunitz-type protease inhibitor	220
Q3S489	Kunitz-type protease inhibitor	221
Q3S482	Kunitz-type protease inhibitor	220
Q3S479	Kunitz-type protease inhibitor	220
Q8H9D6	Kunitz-type trypsin inhibitor	220
Q2XPY0	Kunitz-type protease inhibitor-like protein	213
M1LA62	Kunitz-type protease inhibitor D	219
Q8W126	Kunitz-type enzyme inhibitor S9C11	242
L0AQL9	Kunitz-type proteinase inhibitor PKI2	221
A0A097H120	Clone PI8311 Kunitz-type inhibitor A/B mRNA	203
Q66LL8	Kunitz-type proteinase inhibitor	194
Q66LL2	Kunitz-type proteinase inhibitor group B	194
H9B8J1	Kunitz-type proteinase inhibitor group A1	191
H9B8I9	Kunitz-type proteinase inhibitor group A1	191
Q8LJQ0	Kunitz-type proteinase inhibitor	192
H9B8I8	Kunitz-type proteinase inhibitor group A1	191
H9B8J0	Kunitz-type proteinase inhibitor group A1	190
M1KPH5	Kunitz-type protease inhibitor C	178

Table 2S. Detailed description of the ramped gradient used for LC-MS/MS analysis of potato side stream samples.

Time [mm:ss]	Duration [mm:ss]	Mixture [%B]	Flow [nL/min]
00:00	00:00	5	300
01:00	01:00	5	300
05:00	04:00	12	300
145:00	140:00	37	300
150:00	05:00	50	300
151:00	01:00	100	300
165:00	14:00	100	300
170:00	05:00	5	300

Table 3S: Summary statistics for LC-MS/MS proteomics analysis of potato side stream samples using MaxQuant.

Sample	AKV-Feed	AKV-K2	KMC-Feed	KMC-Food	AKV-PFJ
# total peptides (matched)	1229	1034	1208	3958	7840
# total protein groups	394	334	342	1007	1689
# contaminant proteins	24	26	52	31	41
# reverse protein hits	3	4	3	11	24
# potato protein groups	367	304	287	965	1624
# potential potato proteins	1154	948	912	2356	3633

Table 4S. Identified and quantified patatin isoforms by LC-MS/MS. Proteins were identified by their UniProt AC# and quantified sample-wise using the iBAQ algorithm in MaxQuant (normalized to riBAQ).

AKV-Feed	
**AC#**	**riBAQ**
Q3YJT3	22.6%
Q2MY44	20.2%
Q3YJS9<br>Q2VBJ3<br>Q2VBJ2<br>Q2MYJ6<br>Q2MYJ4<br>Q2MYC5<br>Q2MY85<br>Q2MY98<br>Q2MYF1<br>Q2MYE9<br>Q2MYB8<br>Q2MY88<br>Q2MYB7	4.0%
Q3YJT4<br>Q2MY60<br>Q2MY59<br>Q2MY58<br>Q2MY50<br>Q2VBJ0<br>Q2VBI9<br>Q2VBI6<br>Q2MYY2<br>Q2MYX5<br>Q2MYX4<br>Q2MYW0<br>Q2MYV6<br>Q2MYV1<br>Q2MYT1<br>Q2MYS9<br>Q2MYS7<br>Q2MYS6<br>Q2MYS2<br>Q2MYQ3<br>Q2MYN1<br>Q2MYK2<br>Q2MYJ9<br>Q2MYJ1<br>Q2MYH4<br>Q2MYG9	3.8%

AKV-K2	
**AC#**	**riBAQ**
Q3YJT3	13.7%
Q2MY44	9.3%
Q3YJS9<br>Q2VBJ3<br>Q2VBJ2<br>Q2MYJ6<br>Q2MYJ4<br>Q2MYC5<br>Q2MY85<br>Q2MY88	1.4%

KMC-Feed	
**AC#**	**riBAQ**
P15477	29.6%
Q2MY44	12.5%
Q3YJS9<br>Q2VBJ3<br>Q2VBJ2<br>Q2MYJ6<br>Q2MYJ4<br>Q2MYC5<br>Q2MY85<br>Q2MYB7<br>Q2MY88<br>Q9AUH5<br>M1BFJ1	4.9%
Q3YJT3	4.0%
Q3YJT4<br>Q2MY60<br>Q2MY59<br>Q2MY58<br>Q2MY50<br>Q41487<br>Q2VBJ0<br>Q2VBI9<br>Q2VBI6<br>Q2MYX4<br>Q2MYW0<br>Q2MYV6<br>Q2MYV1<br>Q2MYT1<br>Q2MYS7<br>Q2MYS6<br>Q2MYS2<br>Q2MYQ3<br>Q2MYN1<br>Q2MYK2<br>Q2MYJ9<br>Q2MYG9<br>Q2MYF3<br>Q2MYE1<br>Q2MY96<br>Q2MYM3<br>Q2MYH4	3.8%

KMC-Food	
**AC#**	**riBAQ**
Q2MY60<br>Q2MY59<br>Q2MY58<br>Q2MY50<br>Q3YJT4<br>Q41487<br>Q2MYW0<br>Q2MYK2<br>Q2VBI1	11.2%
Q2MY36<br>Q2MY45<br>Q2MY42<br>P15476	9.3%
Q3YJS9	2.7%
Q3YJT3	1.8%

AKV-PFJ	
**AC#**	**riBAQ**
Q2MY45<br>Q2MY36<br>P15476<br>Q2MY42	12.7%
Q3YJS9<br>Q2VBJ3<br>Q2VBJ2<br>Q2MYJ6<br>Q2MYJ4<br>Q2MYC5<br>Q2MY85<br>Q2MY88<br>Q2MYB7	4.8%
Q2MY44	3.2%
Q3YJT3	1.3%
Q3YJT4<br>Q2MY60<br>Q2MY59<br>Q2MY58<br>Q2MY50<br>Q2MYW0<br>Q2MYK2	1.2%

Q2MYF4<br>Q2MYF3<br>Q2MYE1<br>Q2MY96<br>Q2MYM3<br>Q2MYW8<br>Q2MY72<br>Q2VBI7<br>Q2MYU1<br>Q2MYM8<br>Q2MY67<br>Q2MYW1<br>Q7DMV4	
Q3YJT0	1.6%
Q2VBI2<br>Q2MY99	1.1%
Q3YJT2	1.0%

Q2MYF4<br>Q2MYX7<br>Q2MYW4<br>Q2MYU3<br>Q2MYS5<br>Q2MYP2<br>Q2MYK7<br>Q2MYE8<br>Q2MYW8<br>Q2MY72<br>Q2VBI7<br>Q2MYU1<br>Q2MYM8<br>Q2MY67<br>Q2MYW1	
Q42502	1.3%
P07745<br>M1BFJ0	1.1%

Table 5S. Identified and quantified protease inhibitors (KTIs, PINs, and MCPIs) by LC-MS/MS. Proteins were identified by their UniProt AC# and quantified sample-wise using the iBAQ algorithm in MaxQuant (normalized to riBAQ).

AKV-Feed	
**AC#**	**riBAQ**
Q3S488<br>Q3S482<br>Q3S479	8.8%
Q41484<br>Q2XPX8<br>P58517	5.1%
O24385<br>Q8RX93<br>A0A097H166<br>O24386<br>Q8RX92<br>A0A097H155	3.7%
P58515<br>Q8L5X4<br>M1AKE4<br>Q66LL8<br>Q66LL2<br>Q8L5X3<br>Q8L5X6<br>A0A097H180<br>A0A097H143<br>A0A097H139<br>A0A097H148<br>A0A097H169	2.5%
Q38M77	2.4%
A0A097H164<br>M1AMY0	1.8%

AKV-K2	
**AC#**	**riBAQ**
Q3S493	9.2%
Q41484<br>Q2XPX8<br>Q2XPY0	5.5%
Q3S488<br>Q3S482<br>Q3S479	4.1%
Q38M77	1.7%
Q41432<br>Q3S486<br>O24372	1.6%
P58515<br>P24743	1.5%
Q43648<br>Q07459	1.4%
Q3S485<br>Q3S483	1.3%

KMC-Feed	
**AC#**	**riBAQ**
Q3S488<br>Q3S482<br>Q3S479	8.3%
Q2XPY0	3.4%
P58515	2.3%
Q8H9D6<br>Q00652<br>A0A097H1A0<br>Q8S382	2.2%
H9B8I9<br>P17979<br>P16348<br>P58520<br>P58518<br>H9B8J0<br>M1AKE5<br>K7XKR4<br>A0A097H118<br>M1AKE3<br>P58521<br>C5MRU4<br>Q43646	1.4%
O24385<br>Q8RX93<br>A0A097H166<br>O24386<br>Q8RX92	1.0%
A0A097H158<br>M1AMY0	1.0%

KMC-Food	
**AC#**	**riBAQ**
Q3S488<br>Q3S479<br>Q3S482	12.3%
Q8H9D6<br>Q00652<br>A0A097H1A0<br>Q8S382	7.8%
Q41484<br>Q2XPX8	6.7%
A0A097H164<br>M1AMY0	5.3%
A0A097H157	4.6%
P58515<br>P24743	3.3%
A0A097H120	2.6%
Q38M77	2.2%
O24383<br>M1KPH5<br>Q6PMR6<br>O24384<br>A0A097H155	1.8%
O24385<br>Q8RX93<br>A0A097H166<br>O24386<br>Q8RX92	1.6%
Q3S493	1.1%

AKV-PFJ	
**AC#**	**riBAQ**
Q3S488<br>Q3S479<br>Q3S482	8.9%
A0A097H164	7.5%
Q2XPY0	5.8%
H9B8I9<br>P17979	2.7%
Q8H9D6<br>Q00652<br>A0A097H1A0<br>Q8S382	2.4%
P58515	2.3%
O24383<br>M1KPH5<br>Q6PMR6<br>O24384<br>A0A097H155	2.1%
A0A097H157	2.0%
A0A097H120	1.4%

Table 6S. Identified and quantified proteins in other classes (non-patatin and non-inhibitor) by LC-MS/MS. Proteins were identified by their UniProt AC# and quantified sample-wise using the iBAQ algorithm in MaxQuant (normalized to riBAQ). [1]Probable heat shock-related protein. [2]Cold-stress inducible protein. [3]Probable metallocarboxypeptidase inhibitor. [4]Histone. [5]Fruit-ripening protein. [6]Probable Probable RNA-binding proteins. [7]Eukaryotic translation initiation factor 5A. [8]Probable Kunitz-type inhibitor (KTI-C type). [9]Probable Pathogenesis-related protein. [10]Probable Kunitz-type inhibitor (KTI-C type). [11]Linoleate 9S-lipoxygenase 1. [12]Probable linoleate 9S-lipoxygenase 3. [13]Probable Kunitz-type inhibitor (KTI-C type).

AKV-Feed		AKV-K2		KMC-Feed		KMC-Food		AKV-PFJ	
**AC#**	**riBAQ**	**AC#**	**riBAQ**	**AC#**	**riBAQ**	**AC#**	**riBAQ**	**AC#**	**riBAQ**
M1DZV0	1.3%	M1DJ69	4.3%[1]	M1AMY4	3.1%[8]	M1AMY4	1.8%[10]	Q43189	2.3%[12]
		M0ZT86		M1AMY7		M1AMY7		Q9SAP1	
		M1DH04		M0ZMA9	1.0%[9]	P37831	1.1%[11]	Q9SAP0	
		M1AU55		M0ZMB0				M1AMY4	1.5%[13]
		M1AU51		M0ZMA8				M1AMY7	
		M1AU54		A0A1L1Z6W0					
		M1AU53							
		M1AU49							
		M1AU50							
		O04232	4.0%[2]						
		M1AM40							
		Q6ST32							
		K7X8F1							
		M1A6J5	2.9%[3]						
		M1A6J6							
		M0ZJ50							
		O24639							
		M1BEW2	2.3%[4]						
		M1CM34							
		M1CM33							
		M1AG69							
		M1AG47							
		Q2XPW1							
		M1CUG9							
		M1C576							
		M1CT06							
		M1CUH0							
		Q2XTC9	2.0%[5]						
		M1A8I6							
		M1A8I5							
		M0ZJ84	1.1%[6]						
		Q2VCI6							
		Q38M49							
		M1D6C4							
		M1D7Q3							
		M1D7M9							
		Q38HT3	1.0%[7]						

P56337<br>Q38M78<br>P56335<br>P56333<br>K7VPG0	

Table 7S: Experimental sequence coverage of the selected nine peptides from bioinformatic prediction and *in vitro* functional validation within quantified protein isoforms (patatins, KTI-A, and KTI-B) with 100% identity. Relative abundance (in % riBAQ within each sample) is given for all protein with 100% identity and >90% identity with the respective predicted peptide.

**Sample**	Experimental coverage	Protein Range	**Patatin γ-1**	**γ-36**	**α-10**	**α-12**	**KTI-A β-27**	**KTI-B β-22**	**γ-38**	**γ-40**	**γ-49**
AKV-Feed	None	0%	0.0%	3.8%	0.0%	0.0%	0.0%	0.0%	0.0%	0.0%	0.0%
	Low	< 25%	0.0%	0.0%	0.0%	0.0%	0.0%	0.0%	0.0%	0.0%	0.0%
	Partial	25-75%	0.0%	0.0%	25.3%	0.0%	0.0%	0.0%	5.1%	0.0%	0.0%
	High	>75%	20.2%	0.0%	0.9%	26.2%	0.0%	5.1%	0.0%	5.1%	0.0%
	Full	100%	0.0%	46.5%	0.0%	0.0%	8.8%	0.0%	0.0%	0.0%	0.0%
AKV-K2	None	0%	0.0%	24.5%	0.0%	0.0%	0.0%	5.5%	0.0%	5.5%	0.0%
	Low	< 25%	0.0%	0.0%	0.0%	0.0%	0.0%	0.0%	0.0%	0.0%	0.0%
	Partial	25-75%	0.0%	0.0%	14.5%	14.5%	0.0%	0.0%	5.5%	0.0%	0.0%
	High	>75%	9.5%	0.0%	0.0%	0.0%	0.0%	0.0%	0.0%	0.0%	0.0%
	Full	100%	0.0%	0.0%	0.0%	0.0%	4.1%	0.0%	0.0%	0.0%	0.0%
KMC-Feed	None	0%	0.0%	3.8%	0.0%	0.0%	0.0%	0.0%	0.0%	0.0%	0.0%
	Low	< 25%	0.0%	0.0%	0.0%	0.0%	0.0%	0.0%	0.0%	0.0%	0.0%
	Partial	25-75%	0.0%	0.0%	37.1%	0.0%	0.0%	0.0%	3.4%	3.4%	0.0%
	High	>75%	1.1%	0.0%	0.0%	37.1%	0.0%	3.4%	0.0%	0.0%	0.0%
	Full	100%	42.5%	49.9%	0.0%	0.0%	8.3%	0.0%	0.0%	0.0%	0.0%
KMC-Food	None	0%	0.0%	0.0%	0.0%	0.0%	0.0%	0.0%	0.0%	0.0%	0.0%
	Low	< 25%	0.0%	0.0%	0.0%	0.0%	0.2%	0.1%	0.0%	0.1%	0.0%
	Partial	25-75%	0.0%	0.0%	0.0%	0.0%	0.0%	0.0%	0.1%	0.0%	0.1%
	High	>75%	0.0%	0.0%	13.8%	0.0%	0.0%	0.1%	6.7%	0.1%	0.0%
	Full	100%	11.1%	26.0%	0.0%	13.8%	12.3%	6.7%	0.0%	6.7%	0.0%
AKV-PFJ	None	0%	0.0%	0.0%	0.0%	0.0%	0.0%	0.0%	0.0%	0.0%	0.0%
	Low	< 25%	0.0%	0.0%	0.0%	0.0%	0.0%	0.4%	0.0%	0.4%	0.0%
	Partial	25-75%	0.0%	0.0%	16.3%	16.3%	0.0%	0.0%	0.0%	0.0%	0.0%
	High	>75%	0.7%	0.9%	0.0%	0.0%	0.0%	0.0%	6.2%	0.0%	0.2%
	Full	100%	16.3%	19.6%	0.0%	0.0%	8.9%	5.8%	0.0%	5.8%	0.0%

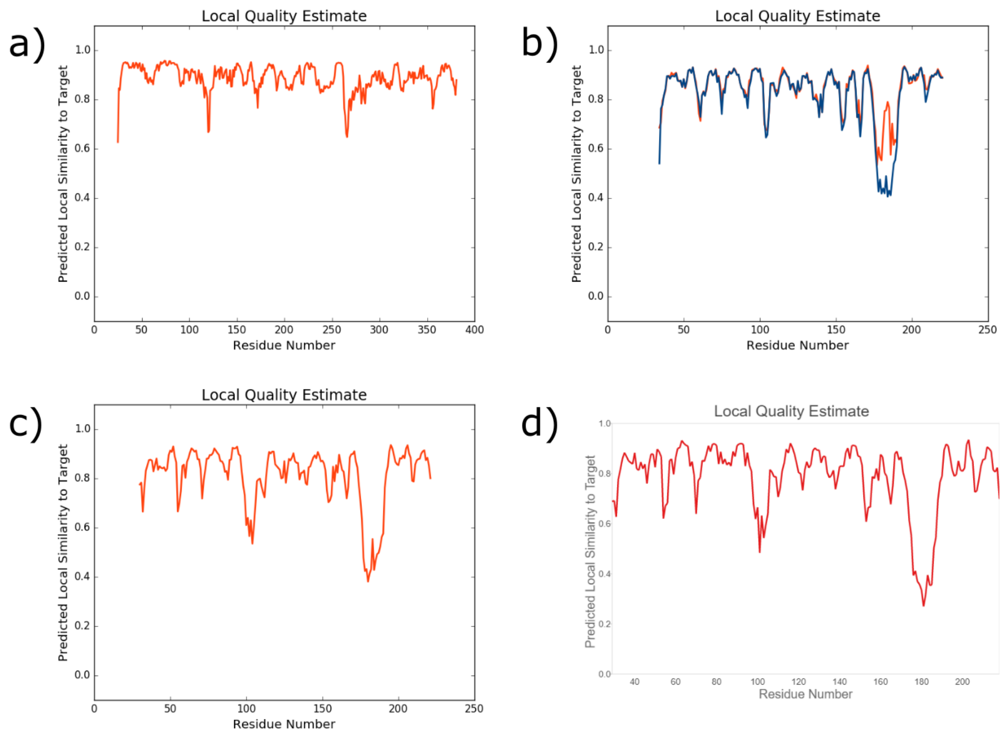

Fig. 1S: Local quality estimates of the template protein models from SwissModel. A) Patatin-B2 (P15477), b) KTI-A (Q3S488), c) KTI-B (Q3S474), and d) KTI-B (Q3S477). QMEAN > 0.75 indicates a good fit between model and template. For KTI-A, QMEAN is shown for both chains in a homo-dimer, where the best fit (chain A, orange) was used for localization of β-27.

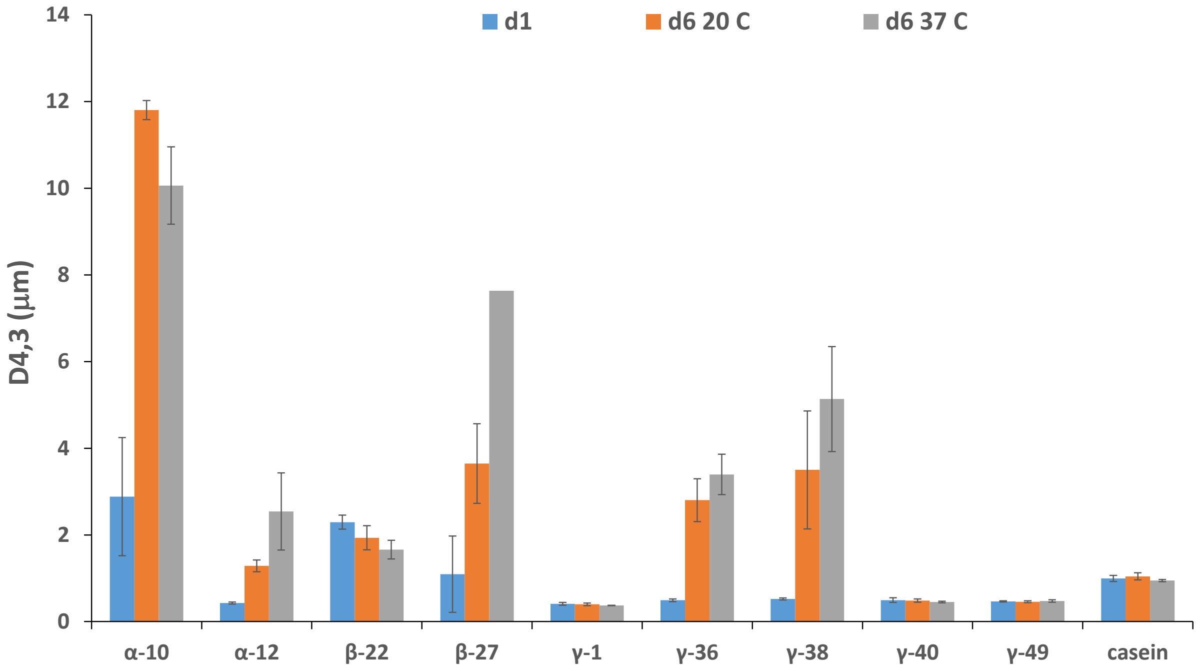

Fig. 2S. Droplet size distribution of emulsions stabilized with selected emulsifier peptides at day 1 and after 6 days storage at 20 °C or 37 °C.
